# Modelling Lithium‐Ion Transport Properties in Sulfoxides and Sulfones with Polarizable Molecular Dynamics and NMR Spectroscopy

**DOI:** 10.1002/cplu.202400629

**Published:** 2024-11-29

**Authors:** Vanessa Piacentini, Cataldo Simari, Emanuela Mangiacapre, Isabella Nicotera, Sergio Brutti, Adriano Pierini, Enrico Bodo

**Affiliations:** ^1^ Department of Chemistry Sapienza University of Rome, P.le Aldo Moro 5 Rome 00185 Italy; ^2^ Department of Chemistry University of Calabria Arcavacata di Rende (CS) 87036 Italy; ^3^ CNR-ISC Consiglio Nazionale Delle Ricerche Istituto Dei Sistemi Complessi Rome 00185 Italy; ^4^ GISEL – Centro di Riferimento Nazionale per i Sistemi di Accumulo Elettrochimico di Energia Florence 50121 Italy

**Keywords:** molecular dynamics, nuclear magnetic resonance, aprotic electrolytes, sulfones, sulfoxides

## Abstract

We present a computational study of the structure and of the transport properties of electrolytes based on Li[(CF₃SO₂)₂N] solutions in mixtures of sulfoxides and sulfones solvents. The simulations of the liquid phases have been carried out using molecular dynamics with a suitably parametrized model of the intermolecular potential based on a polarizable expression of the electrostatic interactions. Pulse field gradient NMR measurements have been used to validate and support the computational findings. Our study show that the electrolytes are characterized by extensive aggregation phenomena of the support salt that, in turn, determine their performance as conductive mediums.

## Graphical abstract

A new polarizable force field for THT and TMS solvents has been parametrized and validated. We used it in molecular dynamics simulations to determine the structural and the transport properties of LiTFSI electrolytes. Our findings are supported by PGF‐NMR diffusional data.

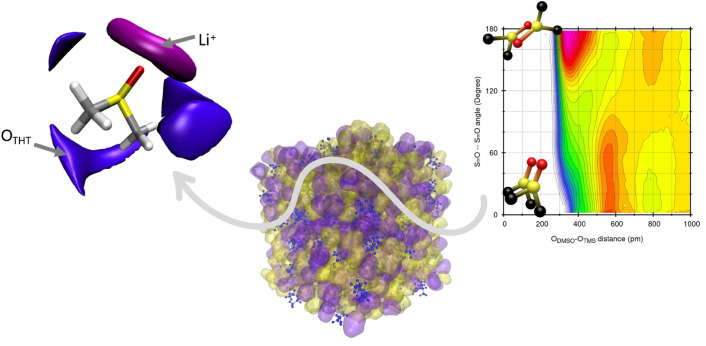

## Introduction

The use of aprotic electrolytes based on organic carbonates, like ethylene carbonate (EC) and propylene carbonate (PC), greatly contributed to the success of Li‐ion batteries^[1]^ (LIBs). Although they settled as the current standard in battery industry, the stability of electrolytes based on carbonate solvents can limit the voltage and temperature ranges of battery operations.[^2^, ^3^]

Sulfur‐based compounds containing S=O (sulfinyl) groups, like sulfoxides and sulfones, are considered as a potential solvent alternative for lithium battery electrolytes. They typically display high dielectric constants, which is important to allow for substantial salt dissociation, but the ionic conductivity can be severely affected by their high viscosity.^[4]^

Among this class of solvents, dimethyl sulfoxide (DMSO) exhibits one of the lowest viscosities, thus providing very good ionic transport. For this reason, as well as for its relatively low toxicity, DMSO has been widely studied as a main solvent in electrolytes for both lithium‐ion and next‐generation lithium batteries (lithium‐sulfur and lithium‐oxygen).[^5^, ^6^, ^7^, ^8^, ^9^] However, the limited stability of DMSO‐based electrolytes is well‐known,[^10^, ^11^, ^12^] thus prompting its use in mixtures with appropriate cosolvents with large electrochemical stability windows.

Sulfone solvents are known to excel in both electrochemical and thermal stability,[^4^, ^13^] but their generally high melting point hinders their use as sole solvent.[^14^, ^15^] Tetramethylene sulfone (TMS, also sulfolane) is by far the most studied in electrochemical applications. Owing to TMS high oxidation potential, which is generally reported to be above 5 V vs Li/Li^+^,^[16]^ its addition to other solvents provides electrolytes with increased anodic stability. Such feature has drawn attention particularly for the possibility to design EC‐free high voltage cells, allowing for cathode materials operating above 4.5 vs. Li/Li^+^,[^17^, ^18^, ^19^, ^20^] as well as for high temperature operating conditions.^[21]^ The addition of TMS can also positively impact the formation of stable and effective passivation layers on the electrodes, as an example in combination with LiBOB^[22]^ and LiFSI[^23^, ^24^, ^25^] salts.

Similarly, tetrahydrothiophene 1‐oxide (THT) is a cyclic sulfoxide with a similar molecular structure of TMS except for the double‐sulfonyl group. THT has been reported to improve the thermal properties and electrochemical stability in PC‐based electrolytes, while also contributing at the same time to a slight increase in ionic conductivity.^[26]^

In this work we employ molecular dynamics (MD) calculations to explore the solvent‐solvent and solvent‐salt interactions and the ion transport in liquid electrolytes based on sulfoxide/sulfone mixtures with 1 mol kg^−1^ lithium bis(trifluoromethanesulfonyl)imide (LiTFSI) salt. The outcomes of the simulations have been validated and compared with a set of conductivity experimental data obtained using both NMR spectroscopy and electrochemical techniques.

Recently, electronic structure calculations have assessed the high stability of sulfones towards electrochemical oxidation and its dependence on intermolecular interactions, suggesting that a decisive role is played by the formation of sulfone‐anion or sulfone dimeric structures.[^27^, ^28^] Also, the properties of sulfolane‐based electrolytes have been investigated with Molecular Dynamics simulations, showing the importance of aggregated or bridged networks in the local structure^[23]^ and their effect on the lithium transport mechanism.[^29^, ^30^] Such kind of investigation, however, has not been extended to understand the complex behavior in mixtures of sulfoxides and/or sulfones.

**Figure 1:**
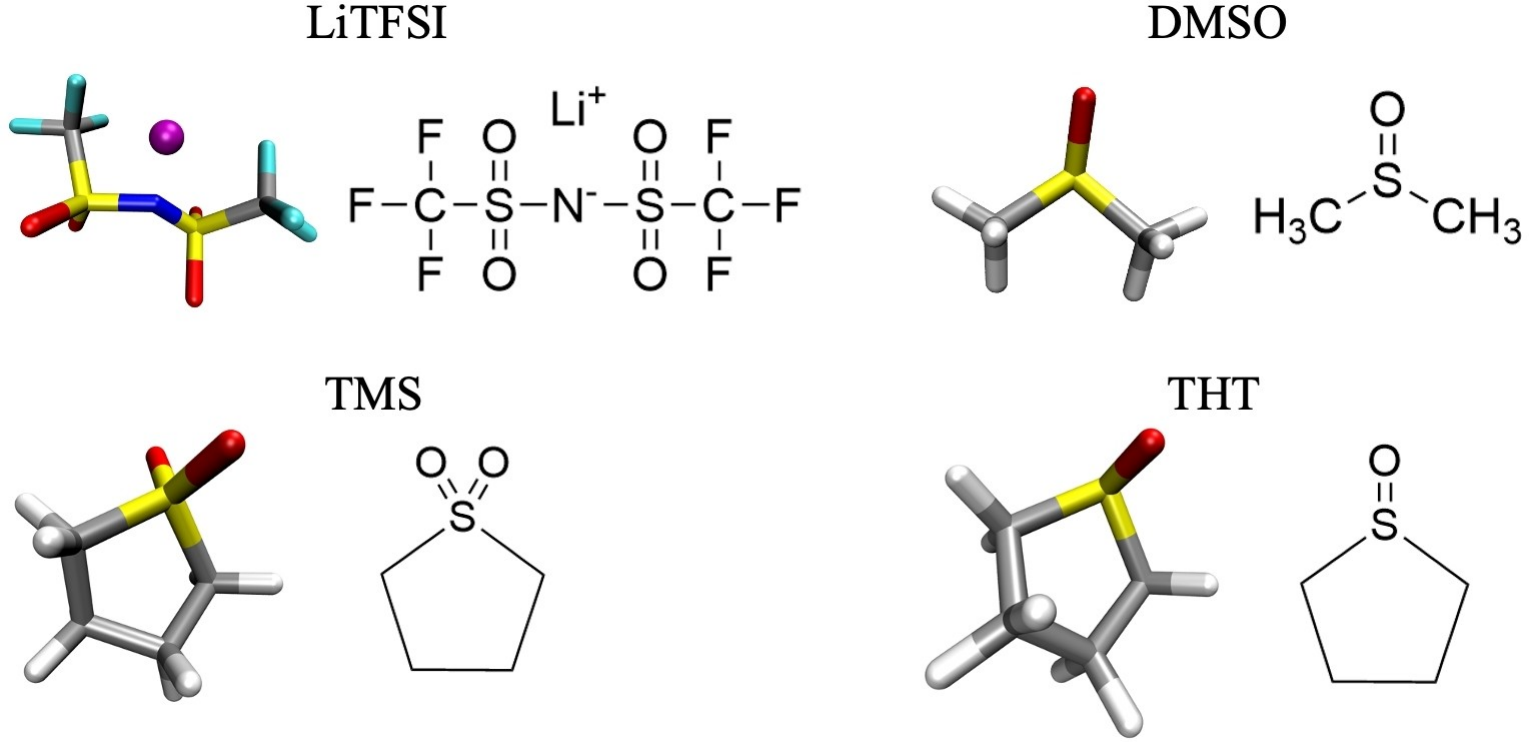
Molecular structures of solvents and salts. In reading order: Lithium bis(trifluoromethanesulfonyl)imide [LiTFSI], Dimethyl sulfoxide [DMSO], Sulfolane [TMS], Tetrahydrothiophene 1‐oxide [THT].

Here, in order to disentangle the roles played by the different compounds inside the electrolyte, an overall set of six different systems were simulated by MD. Three are pure solvents: a DMSO : TMS (50 : 50 v/v) mixture, a DMSO : THT (50 : 50 v/v) mixture and pure THT. The other three are the 1 m LiTFSI solutions of three solvents. Although it possible to obtain a liquid electrolyte at room temperature at some given composition,^[31]^ pure TMS is a solid up to 27 °C, hence pure TMS or TMS/LiTFSI were not simulated. The molecular structures of the solvents and of the salt are reported in Figure 1.

The solvent and electrolyte systems have been investigated using AMOEBA force field model for polarizable molecular dynamics.^[32]^ As thoroughly reported in the literature, the inclusions of polarization into the model potential of MD is highly recommended when simulating concentrated electrolytes.[^33^, ^34^, ^35^, ^36^, ^37^] Moreover, electric polarization has proven to improve the predicted properties of many sulfone‐based electrolytes,^[38]^ which directly applies to the systems of our interest. Conductivities from electrochemical impedance spectroscopy (EIS), transport numbers from potentiostatic measurements and diffusion coefficients from pulse field gradient‐NMR (PGF‐NMR) spectroscopy will also be presented here to assess the quality and reliability of the computational simulations.

## Methods

### Experimental Methods

#### Electrolytes Preparation

DMSO [dimethyl sulfoxide anhydrous, ≥99 %], THT [Tetrahydrothiophene 1‐oxide, 96 %] were acquired from Sigma Aldrich and dried with 3 Å molecular sieves for two weeks before use, whereas TMS [tetramethylene sulfone, Sigma Aldrich, 99 %] and LiTFSI (Lithium bis(trifluoromethanesulfonyl)imide extra dry<20 ppm H_2_O, Solvionic] were used as received. The formulations with the solvents blend were prepared as 50 : 50 v/v. The electrolytes with 1 mol kg^−1^ LiTFSI, were prepared in an argon‐filled glovebox (H_2_O and O_2_ contents<0.1 ppm, MBRAUN).

#### Density

The density was determined using a Mettler Toledo Density Meter DM45 DeltaRange at 293 K. Prior to measurement, a calibration process was performed using dry air and bi‐distilled water at a temperature of 293 K.

#### Conductivities

The electrochemical impedance spectra were recorded at 293 K, using Biologic electrochemical workstation (model VSP) and in the frequency range between 10 kHz and 10 Hz with an applied potential of 10 mV. This analysis enables the determination of the electrolyte‘s resistance value. At elevated frequencies, extracting the bulk resistance value becomes feasible through curve interpolation, utilizing the value derived from the real component of impedance along the abscissa axis. The conductivity is derived via the following equation:[^39^, ^40^] >

(1) $$\sigma =\frac{1}{R}\frac{L}{S}$$

where R is the ohmic resistance, L and S the distance between the blocking electrodes and the electrode area, respectively. The analysis was performance with a coin cell in a symmetric configuration with two stainless steel blocking electrodes (16 mm diameter). A Teflon disc measuring 16 mm in diameter and 2.15 mm in thickness was placed between the two electrodes. The disc was then punched at the center to create a hole with a diameter of 6 mm, into which the electrolyte was inserted.

#### Transport Numbers

The Bruce and Vincent[^41^, ^42^] potentiostatic polarization method was used. The transference number for the cation t_+_ is calculated according to the following equation:

(2) $$t_{+}=\frac{i_{ss}\left(\Delta V-i_{0}R_{0}^{'}\right)}{i_{0}\left(\Delta V-i_{ss}R_{ss}^{'}\right)}$$

where $i_{ss}$ and $i_{0}$ are the steady‐state and initial currents measured by a chronoamperometric test (CA), ▵V is the applied voltage in the CA experiment and 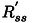 and 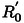 are the electrode resistances after and before the polarization obtained by impedance spectroscopy (EIS). The EIS test has been performed by using a 10 mV voltage bias over a frequency range of 100 kHz to 1 Hz, and for CA an applied potential ▵V=100 mV was used. Measurements were conducted using the ECC std EL‐CELL equipped Whatman® glass fiber separator (1.55 mm thickness, 18 mm diameter) in symmetrical configuration: Li|electrolyte|Li, soaked in 150μl of electrolytes. A BioLogic electrochemical workstation has been used for all tests.

#### Diffusion Coefficients

NMR measurements were conducted using a Bruker AVANCE 300 NMR Wide Bore spectrometer, operating at 300 MHz on ^1^H, 116.6 MHz on ^7^Li, and 282.4 MHz on ^19^F nuclei, respectively. A Diff30 Z‐diffusion 30 G/cm/A multinuclear probe with substitutable RF inserts was employed. Self‐diffusion coefficients and spin‐lattice relaxation times were acquired on ^1^H for DMSO and TEGDME solvent molecules, ^7^Li for Li^+^ cations and ^19^F for TFSI^−^ anions, respectively, at a temperatures of 293 K. To prevent any contact with moisture, the NMR samples were prepared in glovebox and hermetically sealed in 5‐mm Pyrex tubes. For the self‐diffusion coefficient measurements, the pulsed field gradient stimulated echo (PFG‐STE) method was used. The sequence involves three 90° RF pulses (π/2‐τ1‐π/2‐τm‐π/2) and two gradient pulses that are applied after the first and the third RF pulses, respectively. The resulting echo is registered at time τ=2τ1+τm. Following the standard notation, the magnetic field pulses are characterized by their magnitude (g), duration (δ), and time delay (▵). The Fourier‐transformed echo decays were analyzed using the Stejskal–Tanner expression:^[43]^

(3)

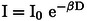




Where the parameters I and I_0_ represent the intensity or area of selected resonance peaks in the presence and absence of gradients, respectively. Meanwhile, β signifies the field gradient parameter, defined as β=[(γgδ)2×(▵‐δ/3)] and D is the measured self‐diffusion coefficient. The experimental parameters employed for the investigated samples were: δ=0.8–2 ms, time delay ▵=8–20 ms, and the gradient amplitude varied from 100 to 950 Gauss cm^−1^. With the low standard deviation observed in the fitting curve and the repeatability of the measurements, the uncertainties in the self‐diffusion measurements are approximately 3 %. Lastly, longitudinal or spin‐lattice relaxation times (T_1_) were determined using the inversion‐recovery sequence (π‐τ‐π/2).

### Computational Methods

The molecular species THT and TMS were parametrized according to the AMOEBA model^[32]^ using the procedure reported in the reference paper of Poltype.^[44]^ The parameters for electrostatic multipoles were fitted on the electrostatic potential calculated from MP2/aug‐cc‐pVTZ electron densities. Atomic polarizabilities, van der Waals, valence and torsion parameters were directly transferred from database matching atomic types. The force field parameters are available in the Supplementary Information. The parameters for DMSO and Li^+^ were taken for the existing AMOEBA database while the TFSI anion was taken from our previous work in ref.  [37] with a variation in one torsional angle (also reported in the SI). For the force field validation, the ab‐initio potential energy curves were calculated with the SAPT2+3 method^[45]^ implemented in the PSI4 package,^[46]^ using an aug‐cc‐pVDZ basis set. The curves were obtained by rigidly displacing the Li^+^ ion from the solvent/salt molecules along an arbitrary direction. The SAPT and AMOEBA energies were computed at the same geometries.

Polarizable molecular dynamics (MD) simulations were performed using the Tinker‐HP^[47]^ software. For each system, a cubic simulation box was first relaxed in NPT ensemble for 5 ns, followed by 5 ns of thermal equilibration in NVT ensembles. The specific compositions of the simulations are reported in Table S1. The production runs were done using the NVT ensembles for 5 ns for the electrolyte systems, while only for 2 ns for the pure solvents, times that were sufficient to achieve convergence of the computed quantities. The Bussi thermostat and Berendsen barostat were used for sampling in NVT/NPT ensemble at a temperature of 293 K and a pressure of 1 bar. The cutoffs for van der Waals interactions and real‐space Ewald summation were chosen to be 12 Å. Bonds terminating in hydrogen atoms were constrained using the Rattle algorithm. The time step was set to 1 fs.

Equilibration times for an electrolyte can be relatively long and mainly affect the state of the support salt, particularly its ionic association degree. Given the timescales, it can be difficult to avoid a bias due to the peculiar choice of the initial configuration. For this reason, two sets of simulations have been done: (i) one set with an initial condition where all LiTFSI ionic couples were associated and distributed randomly in the solvent and (ii) a second set with where initially the salt was entirely dissociated, i. e. where anions and cations were randomly distributed in the solvent. In principle, these two simulations should evolve over time from the two extreme initial configurations and reach a state with the same degree of salt association. Practically, in our case, during the equilibration time, the simulation with initially associated salt evolved showing only a small degree of dissociation, while the one with dissociated starting point evolved toward a significant fraction of associated ionic aggregates. Therefore, we can conclude that in these solvents the LiTFSI salt turns out to be largely associated into ionic couples and clusters thus giving rise to non‐ideal behaviors. Despite the similarity of the final states, the first set of simulations turned out to provide a much better agreement with diffusivity experimental data and, in the following, we will report computational data only from this set.

### Validation of the Force Field Model

An initial validation of the force field has been carried out using experimental densities and by direct comparison with ab‐initio dissociation energy curves between selected pairs. Table 1 reports the computed densities with measured experimental ones. All simulations yielded values within 5 % from the bulk data.

**Table 1:** Density (g⋅cm^−3^) of solvents and electrolytes at 293 K. Comparison between theoretical (MD) and experimental results (exp).

Solvent/solution	ρ (MD)	ρ (exp)
THT	1.14	1.16
THT/LiTFSI	1.20	1.24
DMSO : THT	1.17	1.20
DMSO : THT/LiTFSI	1.19	1.22
DMSO : TMS	1.15	1.14
DMSO : TMS/LiTFSI	1.27	1.27

**Figure 2:**
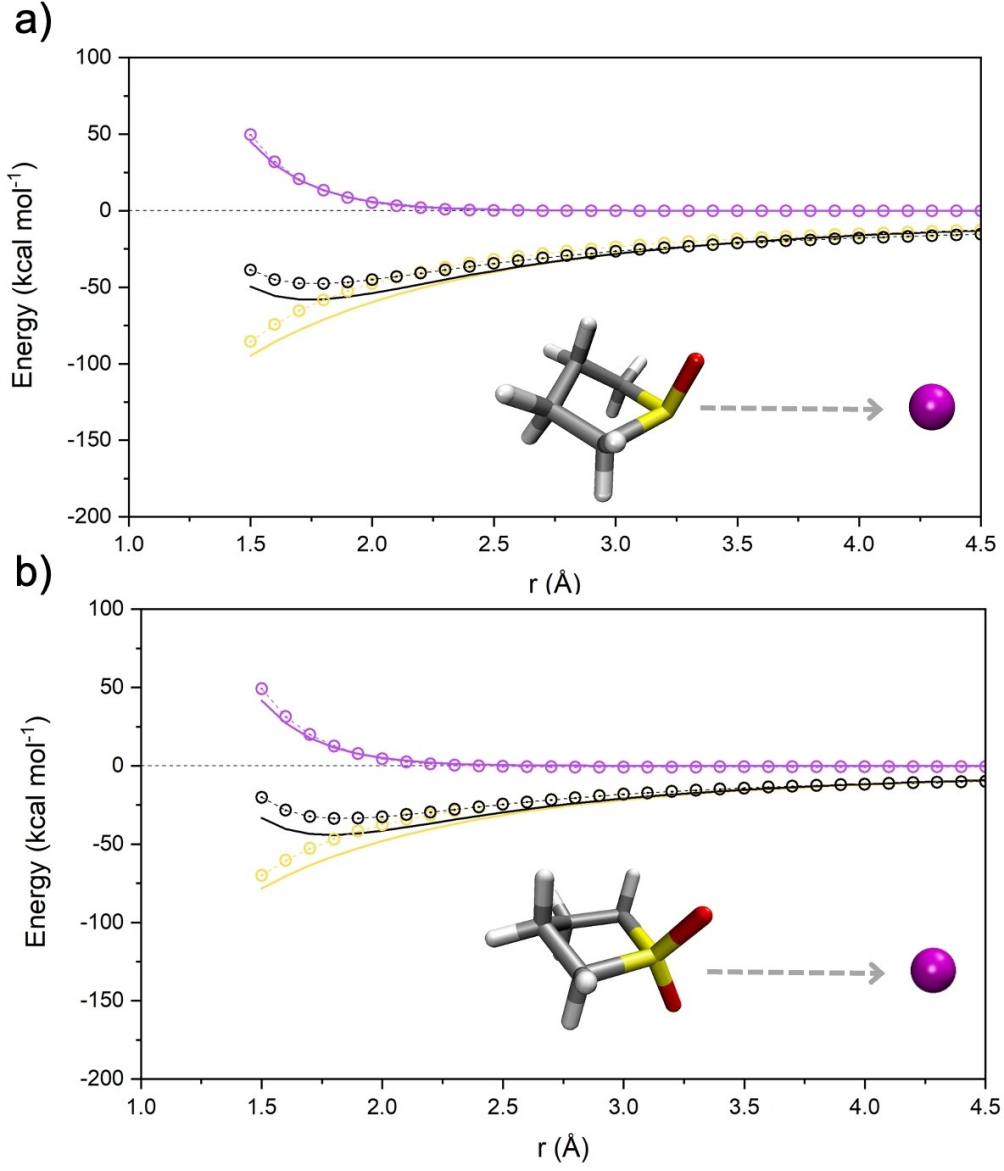
Interaction potential energy curves of a) THT‐Li^+^ and b) TMS‐Li^+^ expressed in kcal mol^−1^. The intermolecular energy calculated with AMOEBA (circles) is compared with ab‐initio SAPT2+3 interaction energy (full lines). The total energy (black) is decomposed in electrostatic (yellow) and van der Waals (violet) contributions.

An example of additional validation is reported in Figure 2 where we have plotted the potential energy curves for the interaction between the two newly parametrized solvent molecules and the Li^+^ ion. The interaction energy computed using a perturbative decomposition analysis of the electronic energy at the DFT level (SAPT) is compared to that stemming from the force field. The sum of dispersion and exchange from SAPT (purple line) is compared to the van der Waals of the latter (purple circles). Electrostatic (including induction) and total energies are reported in yellow and black color, respectively.

The match between the DFT energies and our AMOEBA parametrization is very good over a wide range of distances. Electrostatic appears to be reproduced reasonably well by the force field, except at very short range where electronic quantum effects such as strong polarization and charge transfer take place. Its long‐range behavior is characterized by r^−2^ dependence due to the permanent and induced dipole‐charge interaction. The van der Waals term dies off more quickly (r^−6^) at long‐range but dominates the short‐range region where exchange interactions between electrons become repulsive. Again, the onset of the intermolecular repulsion generated by the force field repulsive terms in the van der Waals potential matches the ab‐initio data.

## Results and Discussion

The understanding of the structural and dynamic properties of a solution phase is crucial to decouple the physic‐chemical parameters that alters the functional properties of any electrolyte. In particular the addition of any salt in a complex solvent mixture leads to a remarkable reorganization of the system, resulting in a variety of structures that do not behave uniformly (i. e. ideally) but contribute differently to the physicochemical properties of the system. Furthermore, the molecules here under study, due to the presence of SO or SO₂ functional groups, create a complex network of intermolecular interactions,[^48^, ^49^] primarily dominated by attractive forces between permanent dipoles. Identifying how these interactions contribute to the formation of local order, or the lack thereof, is crucial for understanding the dynamic and transport properties of liquid systems such as electrolytes.

### Pure Solvents

**Figure 3:**
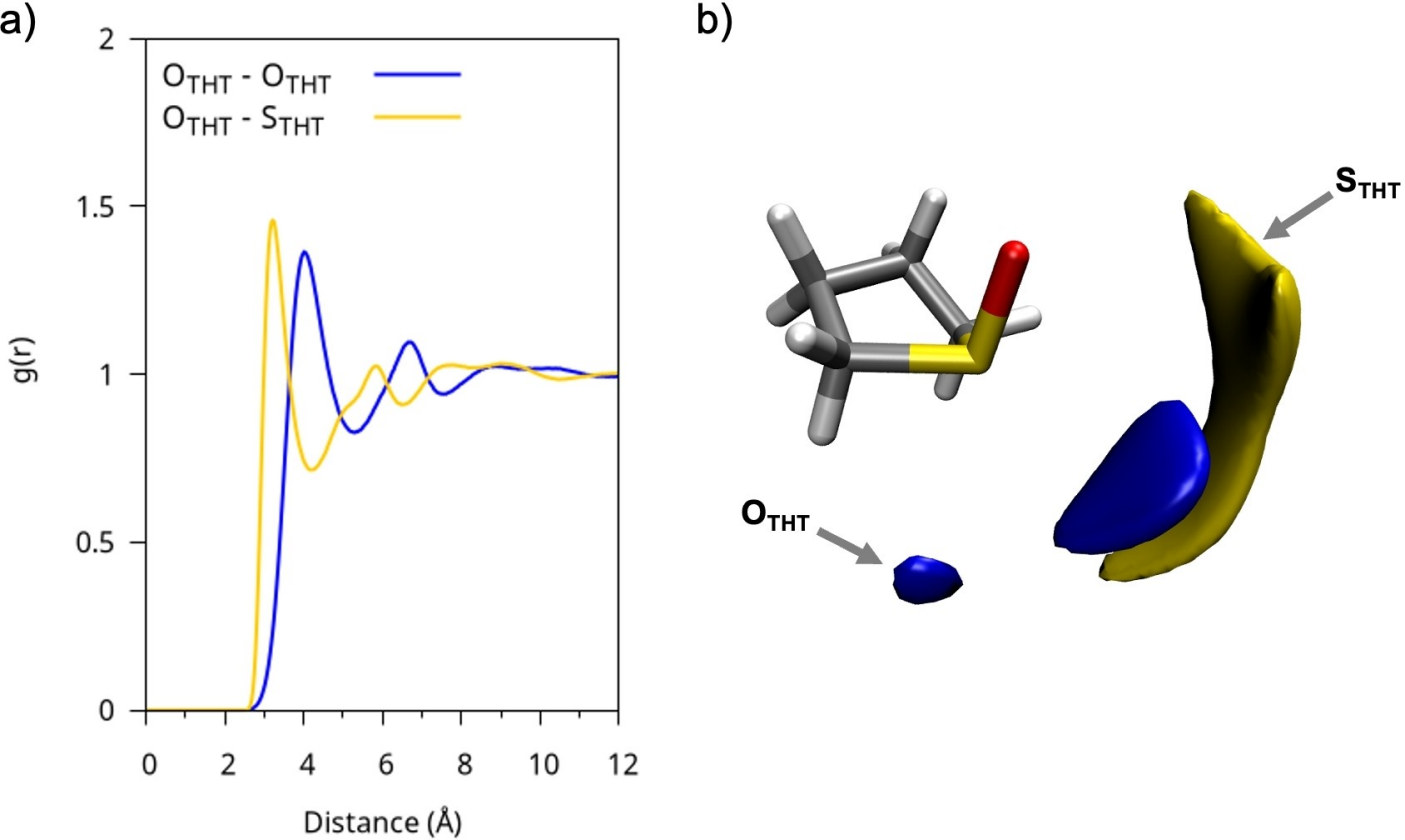
a) RDFs of pure THT between representative pairs of atoms. b) SDFs of the oxygen (blue) and sulphur (yellow) atoms relative to a reference molecule fixed in space.

The interactions between solvent molecules in the bulk phase were investigated using the radial distribution functions (RDFs), selecting O−O and O−S atom pairs both within the same species and between different species of molecules. Starting with the simplest system to describe, the one with THT as the sole solvent (Figure 3), it is easily seen that the short‐range interaction occurs between the oxygen and sulphur atoms of adjacent molecules, where the oxygen atom is oriented toward the lone pair of the sulphur atom (Figure 3a). The arrangement of neighboring molecules stems from electrostatic dipole‐dipole interactions, which dominate the liquid‘s structure, a phenomenon extensively investigated in experimental studies and documented in the literature for similar molecular systems.[^37^, ^50^, ^51^, ^52^, ^53^] The organization of neighboring molecules strongly resembles a head‐to‐tail arrangement, as can be observed through the spatial distribution functions (SDFs) of the O and S atoms around a reference THT molecule (Figure 3b), whose orientation has been fixed in space, with a cutoff applied to the first coordination shell. To investigate this mutual arrangement, a radial‐angular combined distribution function (CDF) is used and reported in Figure S1. On the x‐axis, the distance between neighboring O−O pairs is represented, while the y‐axis shows the angle between the S=O dipoles of neighboring molecules. The CDF in Figure S1 indicates a strong correlation between neighboring THT molecules, with a peak around 4 Å, where the dipoles exhibit a predominantly parallel alignment (~$0^{\circ }$ ), and a smaller but significant fraction of them in an anti‐parallel configuration (~$180^{\circ }$ ).

**Figure 4:**
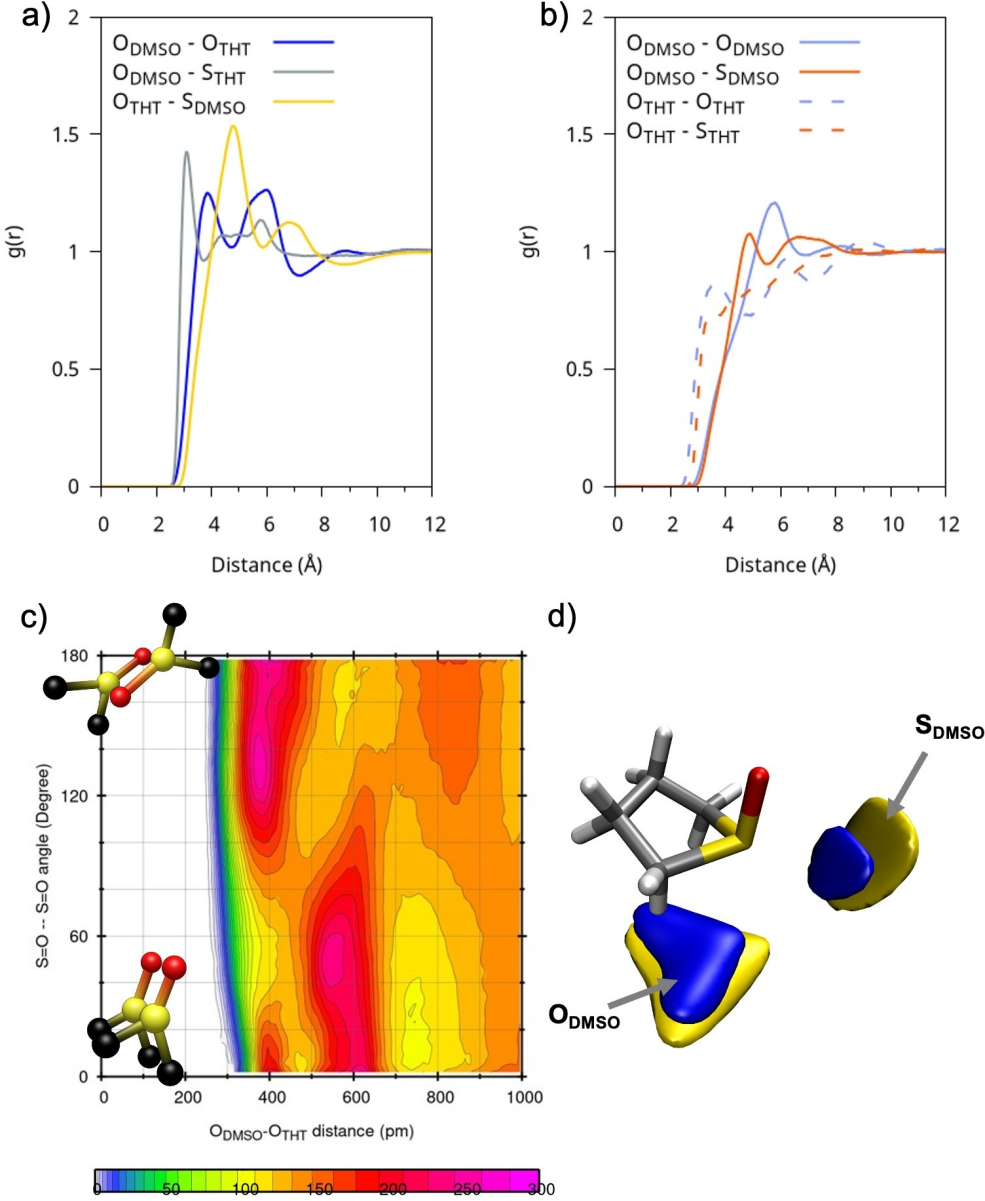
(a) RDFs of the DMSO : THT solution between representative atom pairs between different molecular species; (b) RDFs of the DMSO : THT mix between representative atom pairs between the same molecular species c) Combined radial and angular distribution functions between O−O distances and dipole‐dipole/ S=O−S=O angles of neighboring DMSO‐THT molecules; d) SDFs of oxygen (blue) and sulphur (yellow) atoms in DMSO with respect to a reference molecule of THT.

When mixed with DMSO, THT loses much of its original structure and tends to interact more strongly with DMSO than with itself. This behavior is illustrated in Figures 4a–b, which show the RDFs for O−O and O−S distances for THT/DMSO, THT/THT, and DMSO/DMSO pairs, respectively. The mixing leads to a weakening of intermolecular interactions between THT molecules compared to the pure solvent situation. The main coordination effect in DMSO : THT occurs between the sulphur of THT and the oxygen of DMSO. The relevant RDF is represented by the blue line in Figure 4a, which displays two short‐range peaks: the first, around ~3.8 Å and the second, around ~6 Å. To gain a deeper understanding of these interactions, it is helpful to analyze the CDF shown in Figure 4c. It can be observed that, at distances around 3.8‐4 Å, the interactions are dominated by electrostatic forces, with the dipoles tending to align in an anti‐parallel arrangement, forming an angle of about 150 degrees between them. At greater distances, around 6 Å, a parallel orientation dominates, with an angle of approximately 50 degrees. This reflects the approach of DMSO positioning itself beneath the THT ring as further confirmed by the SDF shown in Figure 4d.

**Figure 5:**
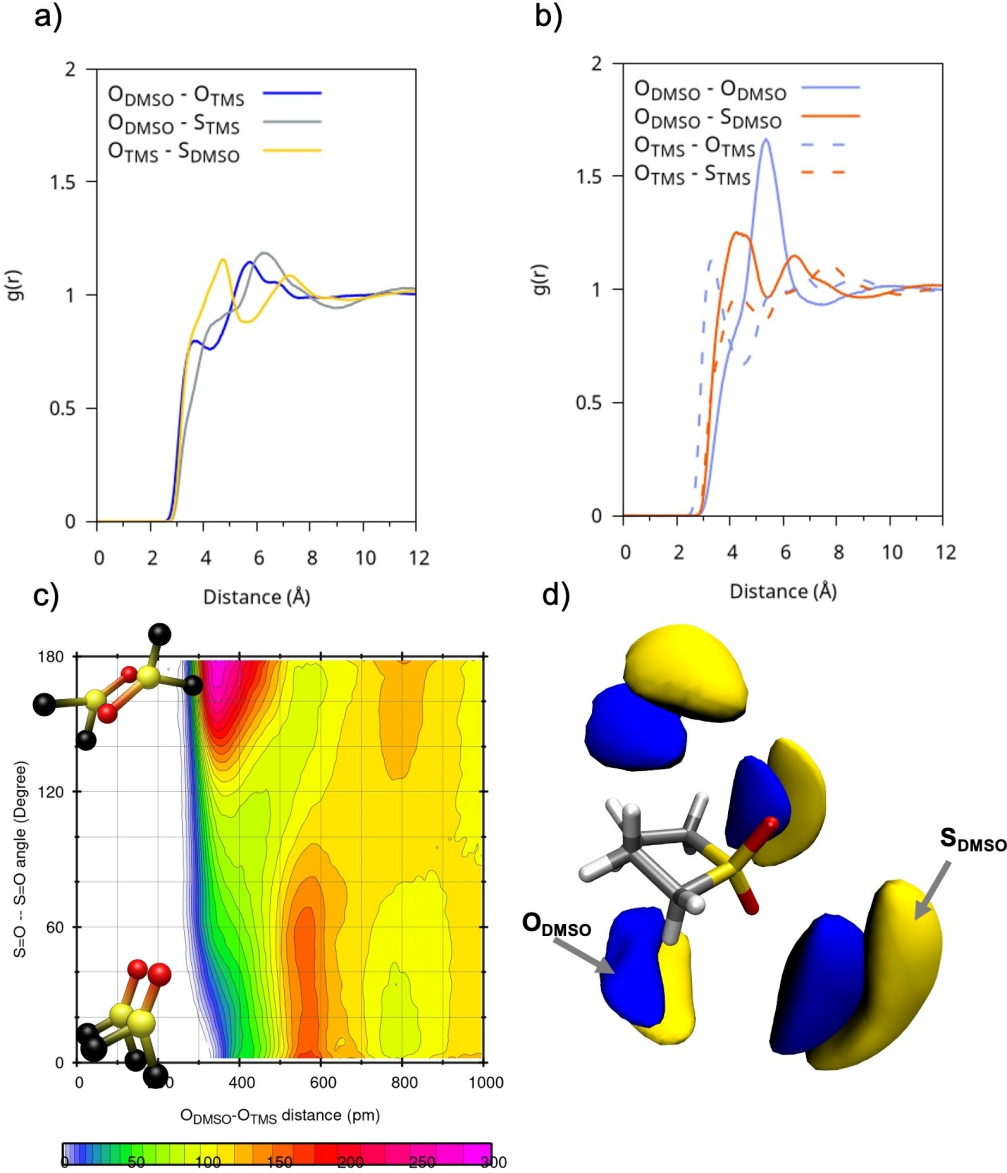
(a) R DFs of the DMSO : TMS solution between representative atom pairs between different molecular species; (b) RDFs of the DMSO : TMS mix between representative atom pairs between the same molecular species; (c) Combined radial and angular distribution functions between O−O distances and dipole‐dipole/ S=O−S=O angles of neighboring DMSO‐TMS molecules; d) SDFs of oxygen (blue) and sulphur (yellow) atoms in DMSO with respect to a reference molecule of THT.

The behavior of the DMSO : TMS mixture differs significantly from that with THT. According to the data shown in Figures 5a–d, which report selected interatomic RDFs, the dominant short‐range interaction, in the mixture, occurs between the homodimer TMS:TMS and DMSO : DMSO species, driven by their strong permanent dipoles (Figure 5b). From the analysis of the DMSO‐TMS CDF (Figure 5c), where the average of all two TMS oxygen atoms was taken, it is observed that the heterodimeric TMS‐DMSO interaction is weaker and non‐directional with the short‐range dipole alignment, at the peak around 3.85 Å, is predominantly anti‐parallel, while at distances around 6 Å, a parallel orientation predominates. As shown by the SDF in Figure 5d, DMSO molecules tend to coordinate TMS more blandly than THT forming a less structured environment. They can either reside near the SO_2_ group along the TMS molecular plane, or they can place themselves near the TMS alkyl portion at larger distances.

An additional set of data for the pure solvents is provided in Table 2 where we report the self‐diffusion coefficients of the molecular centers of mass. The diffusion coefficients, *D*, of solvent molecules were calculated using a fitting of the time dependent mean square displacement (MSD):

(4) $$D=\lim_{t\to \infty }\left\langle MSD\right\rangle =\;\frac{1}{6t}\;\lim_{t\to \infty }\left\langle |r(t\right)-r\left(0\right){|}^{2}\rangle$$

**Table 2:** Diffusion coefficients (D) in 10^−10^ m^2^ s^−1^ for the pure solvents as obtained from simulations (MD) and PFG‐NMR experiments.

	D(DMSO)		D(co‐solvent)	
Solvent	MD	NMR	MD	NMR
THT	–	–	1.7	2.4
DMSO : THT	13	4.5	9.7	3.9
DMSO : TMS	4.6	3.9	3.5	2.9

where r denotes the molecular centre of mass position vector.

We compare the simulated values with those obtained from the NMR measurements. The NMR diffusivity of neat THT and of the DMSO : TMS mixture turn out to be well reproduced, while we make a larger error on the DMSO : THT mixture. We note that a further (empirically‐driven) refinement of the force field parameters would be possible at this point to improve the agreement for DMSO : THT. However, we have decided to keep the broadest compatibility with previous (tested) AMOEBA parameters (e. g. the DMSO ones) and to privilege a non‐specific system approach without incurring in a loss of transferability. As we shall see below, these parametrizations yielded consistent results when applied to the electrolyte systems.

### Electrolytes: Structure and Dynamics

A summary of the results concerning electrolytes is reported in Table 3 where the computed transport properties are compared with their experimental counterparts. This comparison serves two objectives: (i) to validate the reliability and accuracy of the force field and (ii) to provide a solid foundation to consolidate the nanoscopic structural data derived from the MD simulations. One should recall that in PFG‐NMR, the diffusion of all species in solution is measured by tracking specific nuclear signals whereas diffusion coefficients from MD simulations are derived from a linear fit of the MSD(t) of the centers of mass of the molecular species (see eq. (4)). Examples of the MSD(t) function along with its linear fitting are reported in Figure S4 where one can verify how the chosen production time is largely sufficient to converge the diffusional data.

**Table 3:** Diffusion coefficients (D) from NMR and MD in 10^−10^ m^2^ s^−1^; Conductivities (σ) in mS cm^−1^ and Li^+^ transport numbers ($t_{{Li}^{+}}$ ) from MD, NMR and electrochemical measurements (B&V). I.Index is the ionicity index calculated as σ(EIS)/σ(NMR).

LiTFSI@ THT	Specie	D MD	D NMR	σ MD	σ NMR	σ EIS	I.Index	$t_{{Li}^{+}}$ MD	$t_{{Li}^{+}}$ NMR	$t_{{Li}^{+}}\;B\&V$
	Li	0.2	0.3	2.84	3.74	2.30	0.62	0.31	0.37	0.48
	TFSI	0.4	0.5							
	THT	0.6	0.6							

LiTFSI@ DMSO : THT	Specie	D MD	D NMR	σ MD	σ NMR	σ EIS	I.Index	$t_{{Li}^{+}}$ MD	$t_{{Li}^{+}}$ NMR	$t_{{Li}^{+}}B\&V$
	Li	1.1	0.8	13.05	9.59	4.41	0.46	0.41	0.39	0.55
	TFSI	1.6	1.2							
	THT	1.8	1.4							
	DMSO	2.0	1.7							

LiTFSI@ DMSO : TMS	Specie	D MD	D NMR	σ MD	σ NMR	σ EIS	I.Index	$t_{{Li}^{+}}$ MD	$t_{{Li}^{+}}$ NMR	$t_{{Li}^{+}}B\&V$
	Li	1.3	0.7	24.22	12.37	0.71	0.06	0.37	0.38	0.24
	TFSI	2.3	1.1							
	TMS	3.3	1.4							
	DMSO	2.3	1.4							

The agreement between the two sets of data for DMSO : THT and for THT alone are excellent, while we overestimate the experimental value by a factor of two for the DMSO : TMS mixture. Overall experimental and computational estimates are in excellent agreement in consideration to the unavoidable limitation of force‐field based MD to reproduce exactly the frictional properties of fluids. The agreement is even more noteworthy because the force field employed here was not adjusted to yield transport data and was parametrized using ab‐initio inputs for individual molecules.

The total conductivity of the solutions has been computed using the Nernst‐Einstein ideal equation^[54]^ both with the diffusion coefficients from NMR and those from the simulations and compared to the experimental conductivity obtained by EIS. The two experimental conductivities, i. e. σ(NMR) and σ(EIS), are not expected to match in consideration to the BE model assumption of an ideal solution. The ratio of the two numbers (often called “ionicity index”, I.Index= $\sigma_{EIS}/\sigma_{NMR}$ )^[55]^ is however very relevant in as it is a direct estimate of the solution non‐ideality. The non ideality is connected to two possible interpretations: (i) the salt is not in the ideal condition of extreme dilution due to ionic association^[56]^ (LiTFSI is in fact a strongly associated salt in these solvents, see below) and (ii) the cross‐correlation between the motions of +/− ionic pairs is not negligible with respect to the single ion ones (see ref. [57]). This phenomenon occurs because their motions are affected by mutual attractions/repulsions, whereas in an ideal solution, the velocities of the ions are uncorrelated. An electrolyte with an ionicity index of one is ideal, whereas the closer to 0 the larger the non‐ideality.

From the data in Table 2, it is evident that the mixture with TMS has an ionicity index of 0.06, significantly lower than one, especially when compared to THT. Therefore TMS‐based solutions exhibit a highly non‐ideal behavior. We anticipate that this effect is linked to specific structural features of the salt‐solvent interaction. The last three columns of Table 2 report the Li^+^ transference numbers.

Those labeled B&V have been directly measured by electrochemical measurements, while those from NMR and MD are computed using the following expression:

(5) $$t_{{Li}^{+}}=\frac{D_{{Li}^{+}}}{D_{{Li}^{+}}+D_{{TFSI}^{-}}}$$

**Figure 6:**
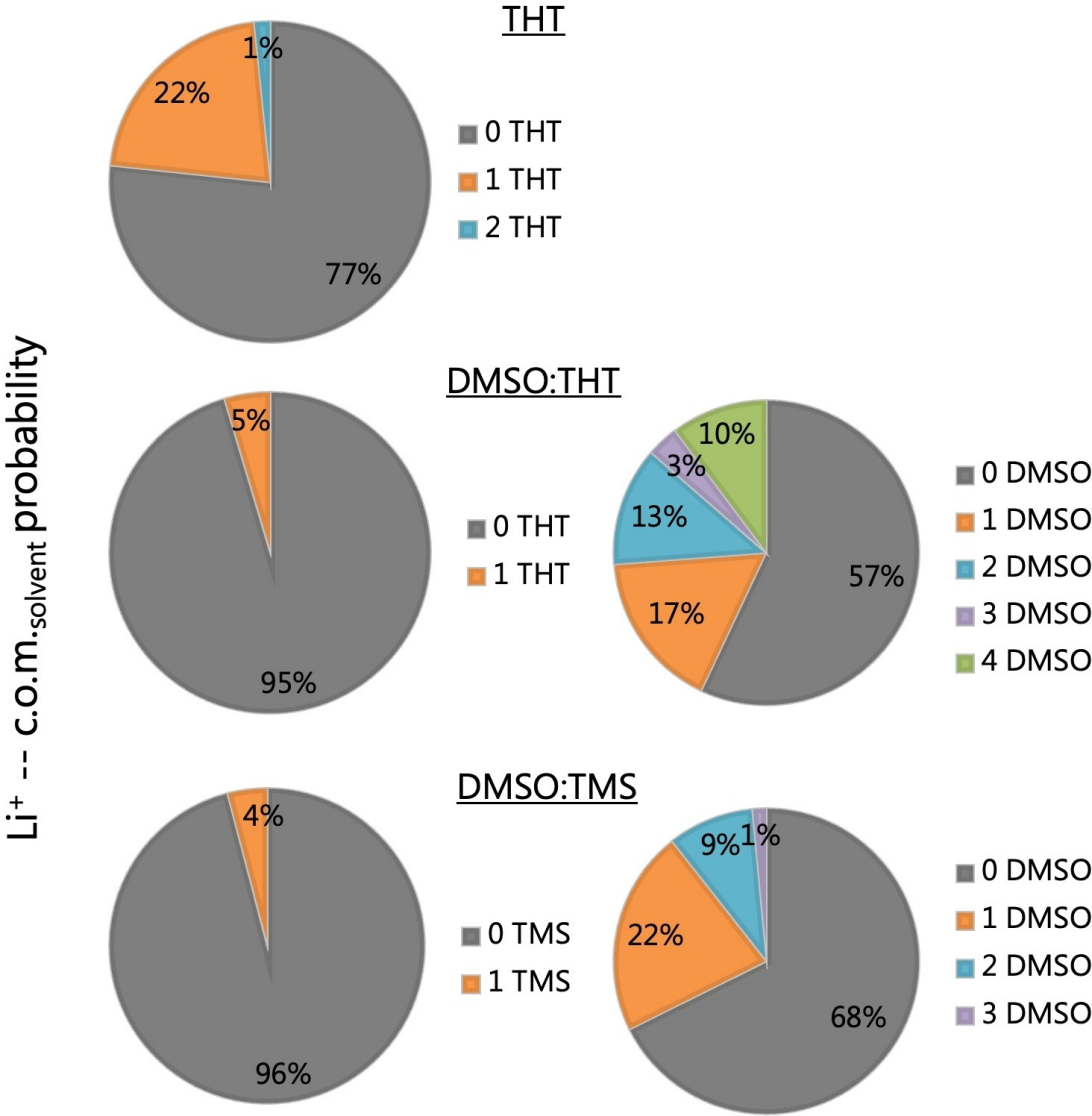
Pie charts quantifying the distribution of solvent molecules around Li. From top to bottom: LiTFSI in THT, LiTFSI in DMSO : THT and LITFSI in DMSO : TMS.

**Figure 7:**
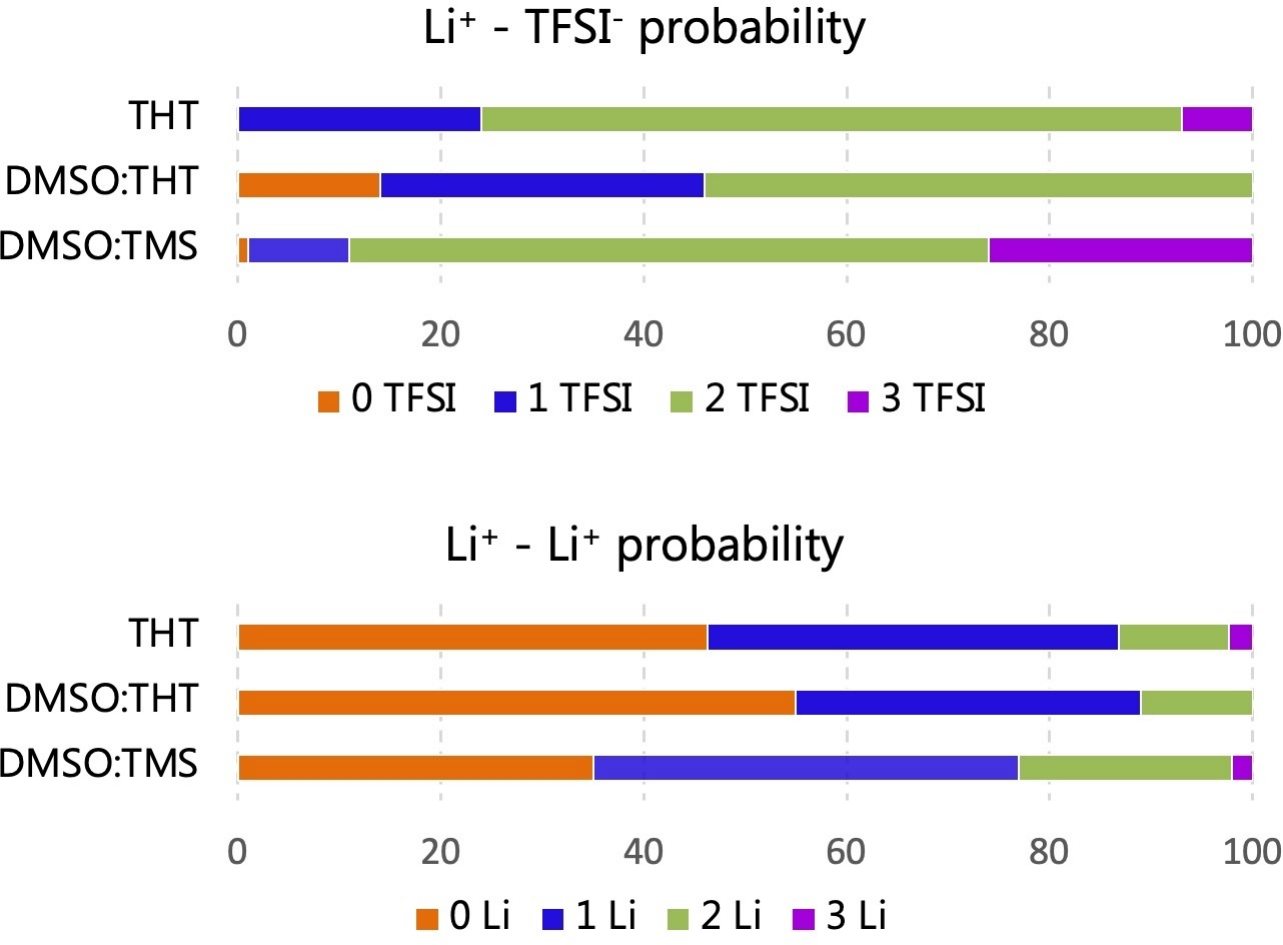
Time‐averaged residence probability of TFSI^−^ anions and Li^+^ inside the first solvation shell of Li^+^ for the three electrolyte formulations.

Since the latter is a ratio, it reduces the differences between the three sets of data which turn out to be quite consistent, because of the removal of any systematic bias. The solvation of lithium in the electrolyte has been analyzed by studying the time‐averaged residence probability of solvent molecules (Figure 6) and ions (Figure 7) in the vicinity of Li^+^. For this analysis, the distance corresponding to the first coordination shell was considered, based on the first minimum observed in RDFs between lithium and the center of mass (c.o.m) of THT, DMSO, TMS and TFSI^−^ (as will be discussed below).

This analysis demonstrates that, although the salt is soluble in all three solutions, the solvents exhibit limited solvating power toward lithium. As shown by the pie charts of Figure 6, only 23 % of Li^+^ cations contain at least one molecule of THT in their first solvation shell. This percentage shrinks to 4–5 % in the two mixtures DMSO : THT and DMSO : TMS, where, however, 40 % and 30 % (respectively) of Li cations have one or more DMSO molecules in their first solvation shells. Overall, Li^+^ cations appear to be only weakly coordinated by the THT and TMS molecules and, when present, it is DMSO that acts as the main neutral solvating molecule.

If we now turn to Figure 7 we see that, examining the probabilities obtained for the LiTFSI@THT system, lithium is always bound to at least one TFSI ion. More surprisingly, most Li cations are actually coordinated by two TFSI ions and few by three. A detailed inspection of the trajectory reveals the formation of Li(TFSI), Li_2_(TFSI)_2_, and Li_3_(TFSI)_3_ clusters.

**Figure 8:**
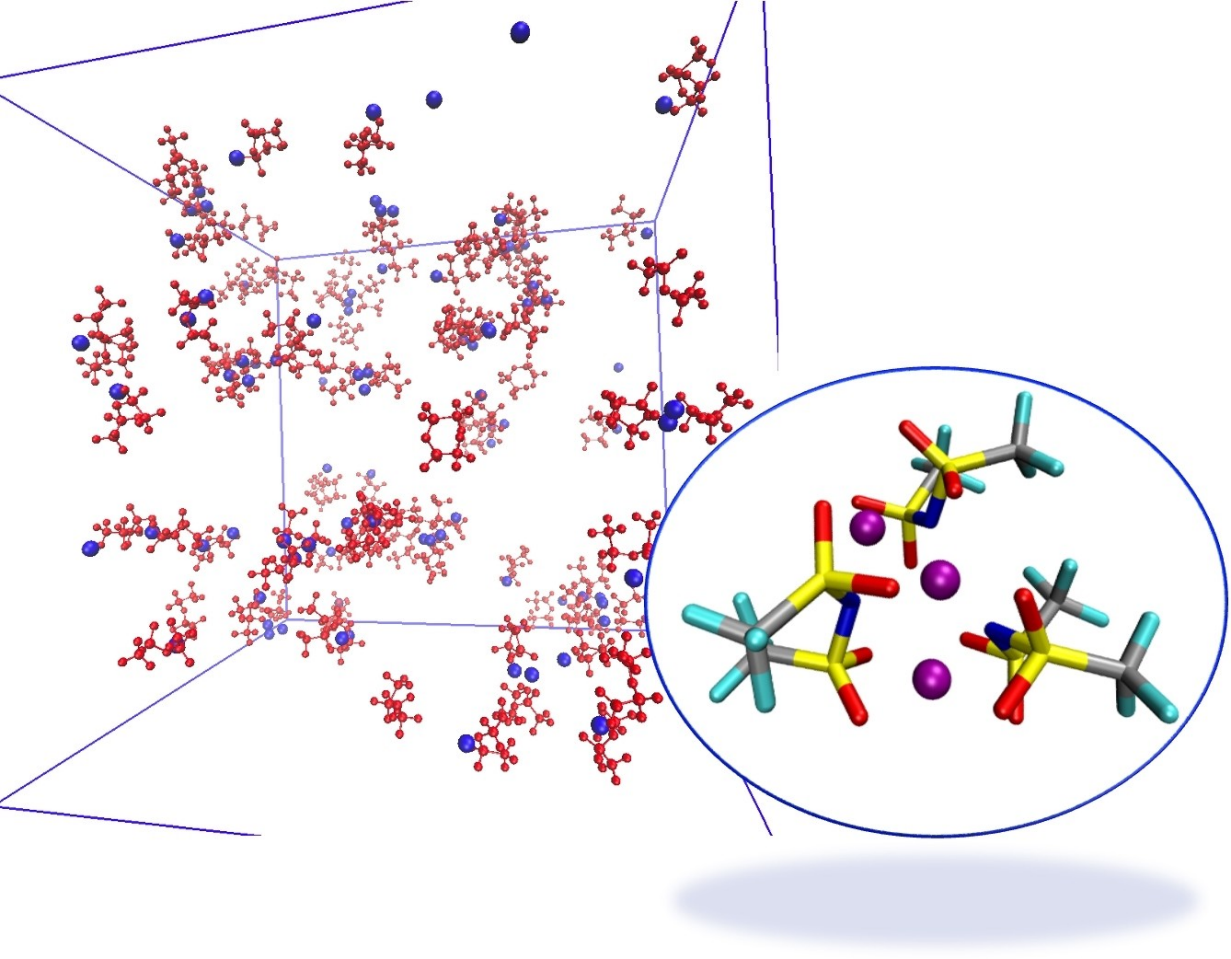
LiTFSI@THT. Left: snapshot of the simulation cell; red structures are TFSI^−^ anions, blue spheres are Li^+^ ions. THT is not shown. Right: example of a Li_3_(TFSI)_3_ cluster.

A representative snapshot of the LiTFSI@THT simulation cell is shown in Figure 8 on the left, clearly highlighting the clustering of the salt.

The presence of (LiTFSI)_n_ cluster has also been noted before in other context,^[58]^ but also when LiTFSI is dissolved in organic carbonates.^[59]^ To further verify that these unusual cluster shapes were not the result of an artefact of the simulation we checked the existence of such structures using a DFT optimization. The computational evidence that these structures are indeed minima over the potential energy surface of (LiTFSI)_n_ is shown in the SI, figure S2. This evidence is further strengthened by the negative formation energies and by the near zero solvent‐exchange ones reported in Table S2.

It is worth mentioning at this point the analogous set of simulations (set (ii) in the computational methods section) where all salt was entirely dissociated at the beginning of the simulation, yielded value of the diffusion coefficients that were consistently farer from the experimental points than those presented in Table 2 (they are in Table S3). Evidently, an initial configuration that assumes a fully dissociated system represents a state of the fluid that is far from the real one. This computational evidence is an additional indirect proof that salt aggregation noted in the structural analysis is not a mere byproduct of the computational model.

**Figure 9:**
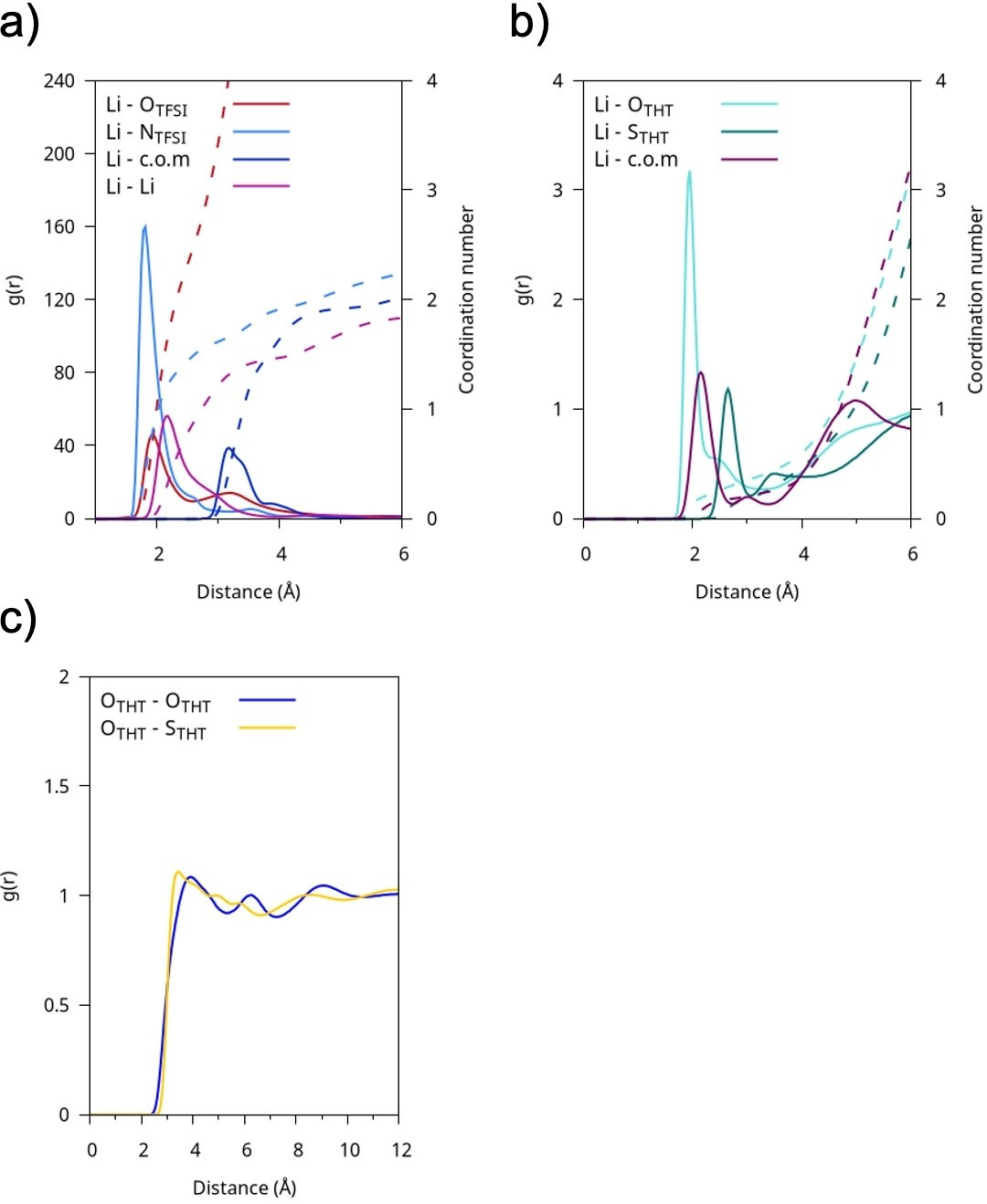
LITFSI@THT: RDFs (g(r)) of a) Li−O(TFSI), Li−N(TFSI), Li‐c.o.m(TFSI), Li−Li and b) of Li−O(THT), Li−S(THT), Li‐c.o.m(THT); the respective cumulative volumetric integrals (coordination number) are shown with dashed lines (scale on the right). c) RDFs of O−O and O−S between THT molecules.

A more quantitative view of the LiTFSI@THT electrolyte structure is given in Figure 9, where the RDFs between the lithium and ions and solvent molecules are shown. The strongest correlations are those of Li^+^ with TFSI^−^ and other Li^+^ ions, while the interaction with the oxygen of THT is only marginal. The picture that emerges is that each Li ion is coordinated by more than one TFSI^−^ (with coordination number rising to 3.5 for oxygen and 2 for nitrogen) and an average of about 1.5 Li^+^. This arrangement is not surprising since it is also the coordination pattern in solid LiTFSI,^[60]^ where two TFSI anion coordinate a close pair of Li ions encaging each cation in a tetrahedral oxygen structure. This behavior is further explained by the preference of the TFSI^−^ anion for a cisoid conformation, which favors short‐range interactions between lithium and the nitrogen of the anion at approximately 1.8 Å, a distance that is shorter than the one with oxygen. This conformation has been observed in several experimental and computational studies when the anion coordinates small cations such as Li^+^ or Mg^2+^.[^61^, ^62^, ^63^]

Slightly more surprising is the appearance of Li_3_(TFSI)_3_ units as the one shown in Figure 8 on the right. It must be taken into account that, in polarizable simulations, the induced dipoles can screen the bare ion‐ion electrostatic repulsion very effectively, thus (realistically) improving the stability of apparently counterintuitive ionic arrangements.

**Figure 10:**
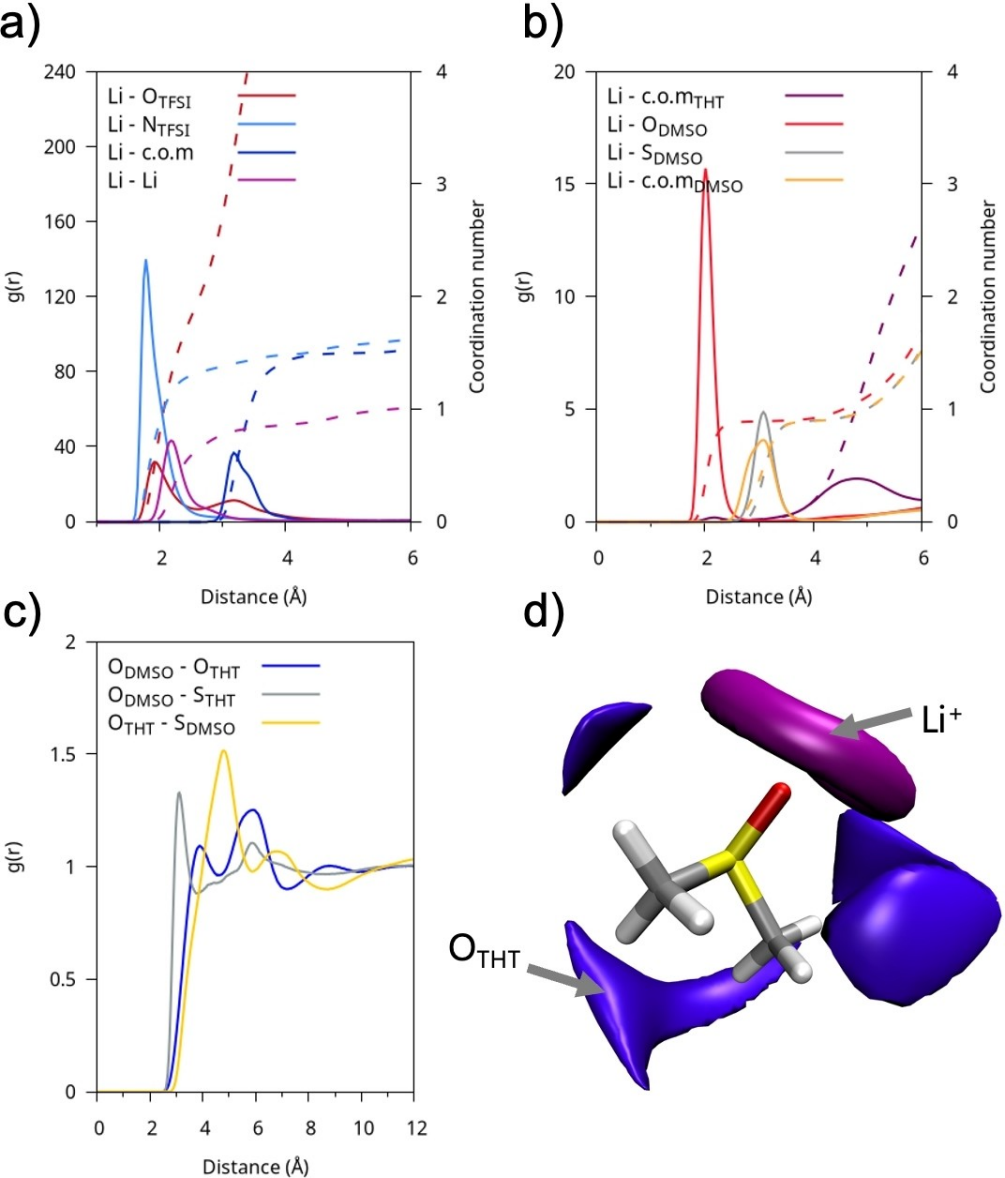
LITFSI@DMSO : THT: RDFs (g(r)) of a) Li−O(TFSI), Li−N(TFSI), Li‐c.o.m(TFSI), Li−Li and b) of Li‐c.o.m(THT), Li−O(DMSO), Li−S(DMSO), Li‐c.o.m(DMSO); the respective cumulative volumetric integrals (coordination number) are shown with dashed lines (scale on the right). c) RDFs of O−O and O−S between DMSO and THT molecules; d) SDFs of lithium (purple) and oxygen atom of THT (violet) with respect to a reference molecule of DMSO.

Examining the LiTFSI@DMSO : THT electrolyte, we observe that the addition of DMSO improves solvation and promotes the separation of ion pairs, resulting in 14 % of Li^+^ ions not coordinated to the counterion, compared to the complete association observed in the previous system (Figure 7). The graphs in Figure 6 show that THT acts primarily as a spectator, without significantly interacting with Li^+^, while DMSO contributes to its coordination with a probability of 43 %. This situation is quantified by the Li‐anion, Li−Li, and Li‐solvent RDFs shown in Figure 10. Compared to the system with only THT, the Li−Li interaction, due to the formation of Li_n_(TFSI)_n_ clusters, is less predominant, with an average coordination number for Li^+^ of about 0.86, indicating a preference for the Li₂(TFSI)₂ configuration. The coordination number of DMSO, equal to 1, also highlights the presence of significant interactions between Li^+^ and the O=S of DMSO.

**Figure 11:**
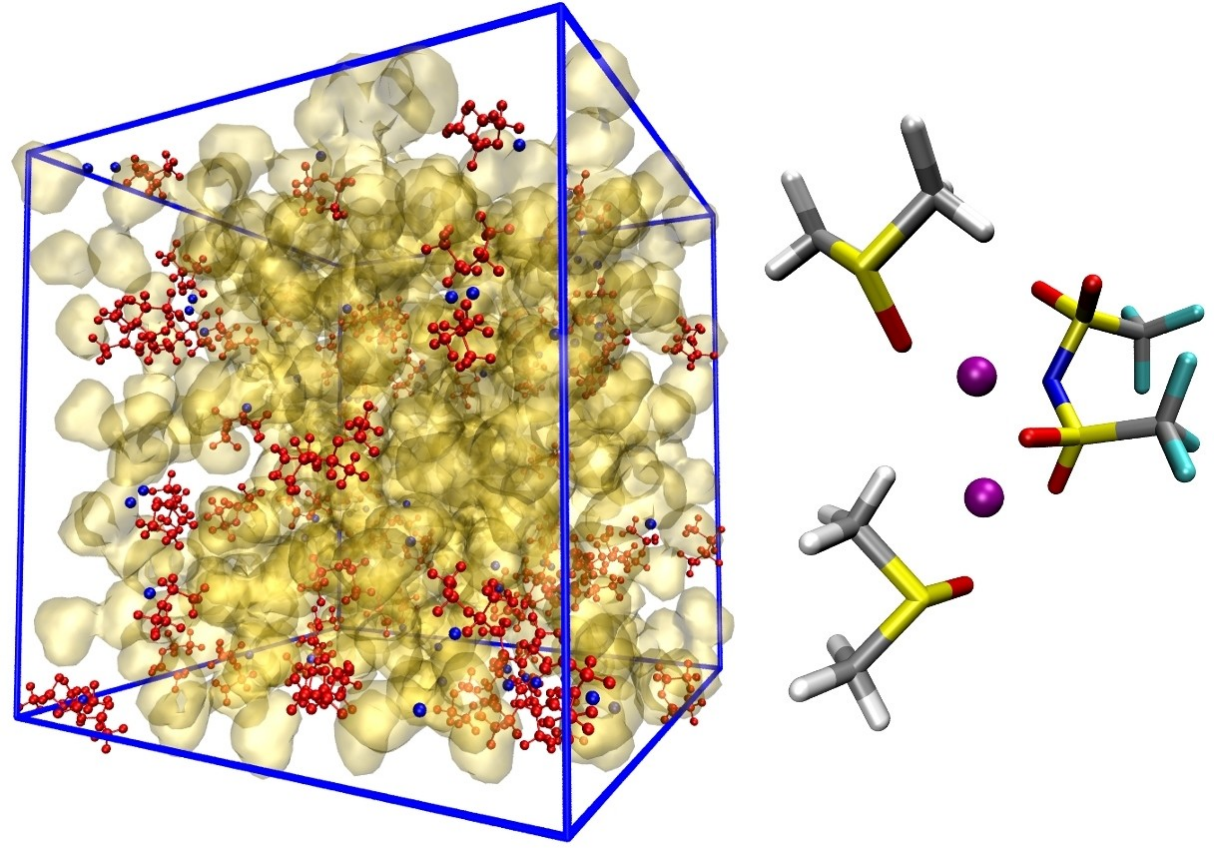
LiTFSI@DMSO : THT: Left: snapshot of the simulation cell. DMSO is shown as a yellow surface, THT is not shown, TFSI anions are shown in red and lithium ions in blue. Right: example of a Li_2_(TFSI)(DMSO)_2_ cluster.

The structure of the electrolyte is shown in Figure 11 using a snapshot of the simulated system, in which the salt structures including the aforementioned [Li(TFSI)]_n_ clusters with n=2,3 are highlighted. Although the salt primarily interacts with itself, a closer inspection of the trajectory reveals that the anions show some affinity for THT (see Figure S3); however, it is DMSO that participates in the solvation of LiTFSI aggregates through S=O−Li^+^ coordination, as shown in the right panel of Figure 11.

The third system is LiTFSI@DMSO : TMS, which includes sulfone (SO₂ group) molecules. The presence of ′free′ anions (and hence dissociated salt) is lower than in the DMSO : THT system, amounting to around 1 % (Figure 6, bottom row), with the formation of [Li(TFSI)]_n_ clusters. In this case as well, the co‐solvent, TMS, does not play a significant role in the coordination of lithium.

**Figure 12:**
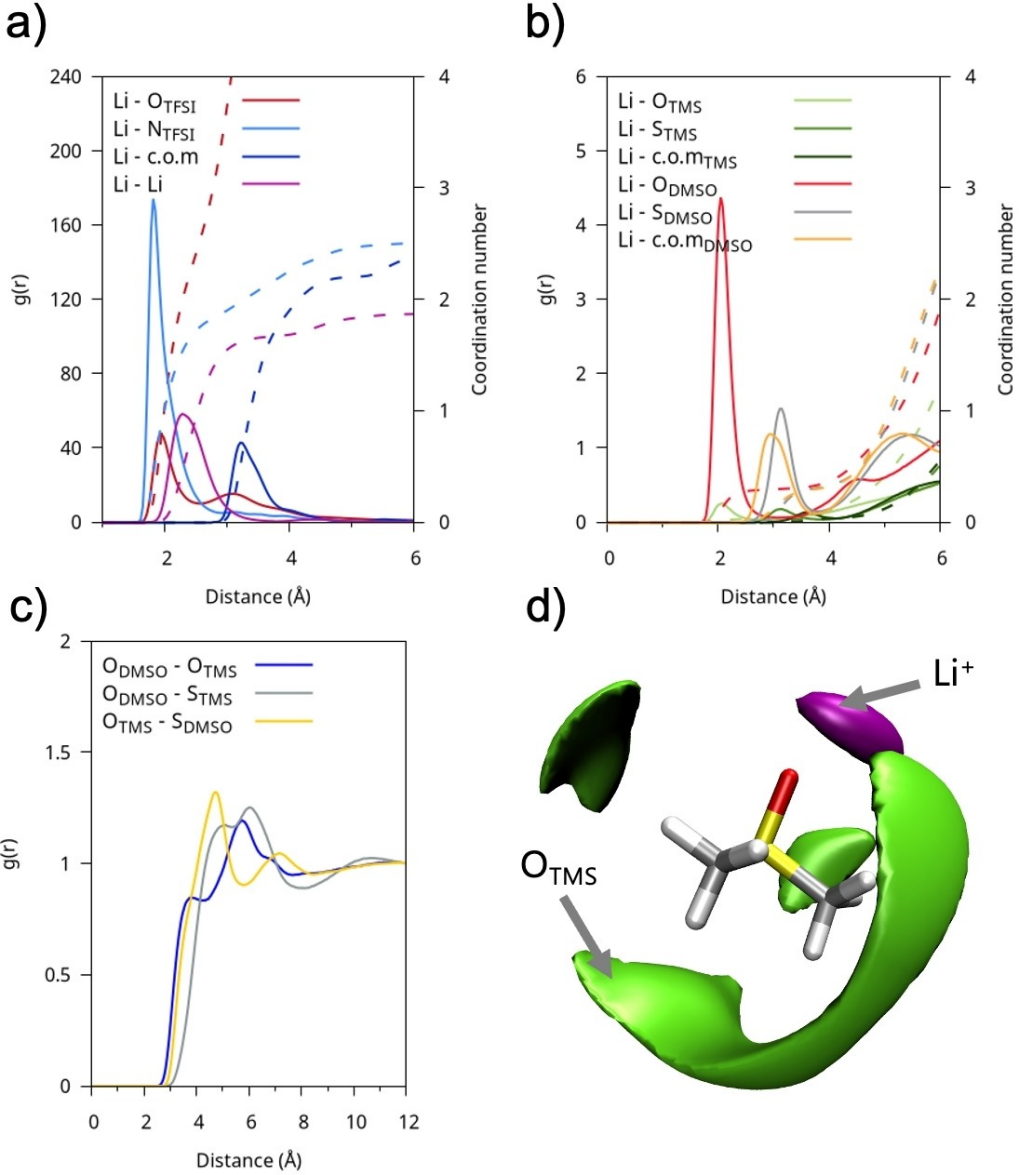
LITFSI@DMSO : TMS: rdfs (g(r)) of Li−O(THT) (green), Li−O(TFSI) (blue), Li−O(DMSO) (yellow) and Li−Li (grey). The respective cumulative volumetric integrals are shown with dashed lines (scale on the right).

**Figure 13:**
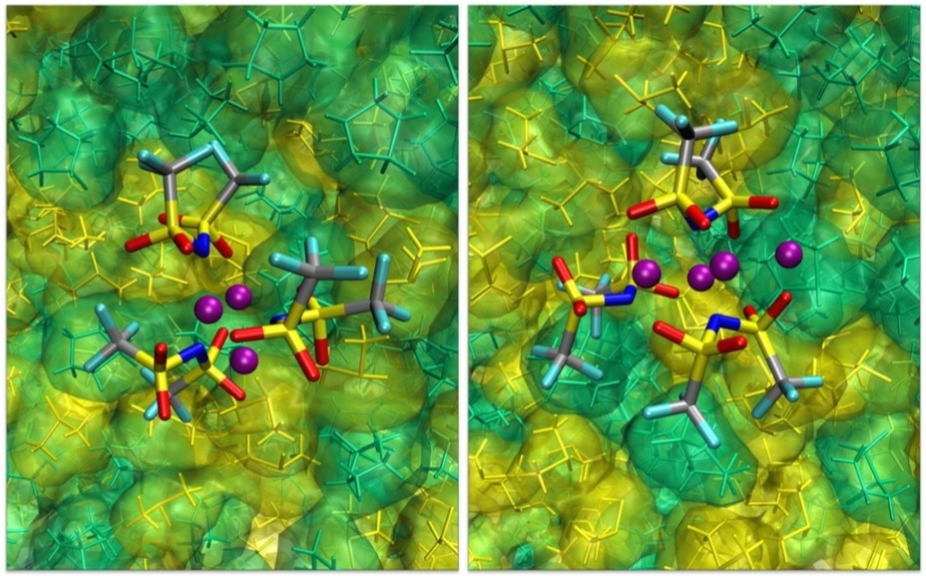
Examples of a Li_3_(TFSI)_3_ (left) and of a Li_4_(TFSI)_3_ cluster embedded in the DMSO : TMS environment. TMS is mapped in green and DMSO in yellow.

Structural information from the RDFs, as shown in Figure 12, reveals that unlike the DMSO : THT mixture, DMSO here plays a more ancillary role, with only weak interactions with Li^+^. As before, the organic component (TMS) remains a spectator with no noticeable ability to interact with the cations, while strong dipolar interactions between solvent molecules are still evident (see figure 12c–d). As mentioned earlier, cluster formation is more prominent in the TMS mixture compared to THT (see Figure 7), indeed the average coordination number for Li−Li interactions is nearly 2, indicating a prevalence of large structures that involve (Li)_n_ moieties. Examples of these structures are shown in Figure 13.

## Conclusions

This work presents a new FF based on the AMOEBA paradigm, designed for two solvents that are gaining attention in lithium battery electrolyte research. In this FF, the interatomic electrostatic potential is described by a multipole expansion that includes static quadrupoles and induced dipoles. The inclusion of many‐body terms in the multipole expansion allows for a consistent and accurate description of the electrostatic forces, which is particularly important in systems with high salt concentrations where polarization plays a significant role. The force field was validated by comparing potential energy curves derived from ab‐initio calculations and has been used to provide both the local molecular structure and the dynamics of the electrolytes. The agreement with ion mobility experimental data was satisfactory, and a detailed analysis of the structural characteristics of the electrolytes at the molecular level was presented, with particular attention to the solvation structure of Li^+^. This analysis has revealed that the presence of DMSO favors salt dissociation and that DMSO is directly involved in lithium solvation. However, despite the high polarity of these solvents, they tend to have a limited dissociative effect on LiTFSI, resulting in a notable prevalence of ion pairs and larger aggregates in solution. The most notable configuration involves two TFSI anions surrounding one or two lithium ions. This means that lithium ions may have a TFSI bridging their coordination spheres or be trapped in a cage formed by the S=O groups of the anions. This effect appears to stem from the preference of solvent molecules to interact with each other through dipole‐dipole interactions in parallel or anti‐parallel configurations. The formation of salt ion pairs or aggregates is not a new phenomenon and has been previously observed in other electrolytes based on carbonates and glyoxal acetals.[^36^, ^64^]

The presence of a co‐solvents in addition to DMSO reduced the extent of lithium solvation compared to pure DMSO, leading to the incorporation of TFSI into the lithium coordination shell. This could contribute to the formation of LiF‐rich interfaces^[65]^ between the electrode and the electrolyte, which are known to inhibit lithium dendrite growth and accommodate the significant volume changes of high‐voltage or high‐capacitance electrodes.

## Conflict of Interests

The authors declare no conflict of interest.

1

## Supporting information

As a service to our authors and readers, this journal provides supporting information supplied by the authors. Such materials are peer reviewed and may be re‐organized for online delivery, but are not copy‐edited or typeset. Technical support issues arising from supporting information (other than missing files) should be addressed to the authors.

Supporting Information

## Data Availability

The data that support the findings of this study are available from the corresponding author upon reasonable request.
