# Supplementary material for: Modelling Lithium‐Ion Transport Properties in Sulfoxides and Sulfones with Polarizable Molecular Dynamics and NMR Spectroscopy
Source: Chempluschem. 2024 Nov 29;90(2):e202400629. doi: 10.1002/cplu.202400629 (PMC11826135; doi:10.1002/cplu.202400629)
Supplement: Supplementary file 1 — Supporting Information [file CPLU-90-e202400629-s001.pdf]

ChemPlusChem

Supporting Information

Modelling Lithium-Ion Transport Properties in Sulfoxides and Sulfones with Polarizable Molecular Dynamics and NMR Spectroscopy

Vanessa Piacentini, Cataldo Simari, Emanuela Mangiacapre, Isabella Nicotera, Sergio Brutti, Adriano Pierini,* and Enrico Bodo*

Table S1. Composition of the simulation cells.

	N° MOLECULES	N° IONS	N° ATOMS
THT	900	-	12600
THT/ LITFSI	900	94	14010
DMSO:THT	600 DMSO+473 THT	-	12622
DMSO:THT/ LITFSI	600 DMSO+473 THT	80	13822
DMSO:TMS	600 DMSO+447 TMS	-	12705
DMSO:TMS/ LITFSI	600 DMSO+447 TMS	88	14025

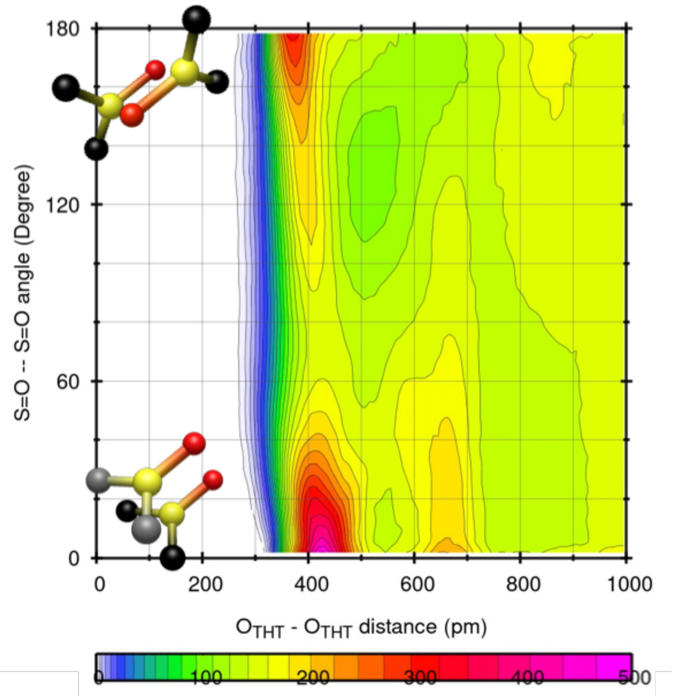

Figure S1. Combined radial and angular distribution functions between O-O distances and dipole--dipole/ S=O--S=O angles of neighboring THT molecules.

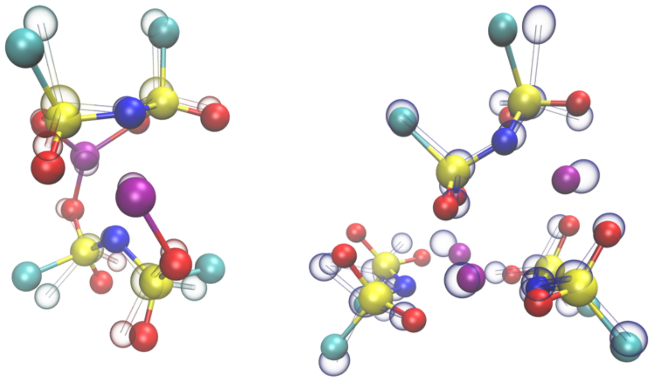

(LiTFSI)$_2$ RMSD=0.703 A (LiTFSI)$_3$ RMSD=0.723 A

Figure S2. Comparison of the AMOEBA in-vacuo minimum energy structures with those obtained from the same starting geometry using the DFT method B97-3c. The full color structure is the AMOEBA geometry, the transparent one is the B97-3c calculation. The RMSD between them is also reported.

Table S2. Formation and exchange energies of ionic clusters calculated with the B97-3c method in CPCM.

	ΔE [kcal/mol]
Li_2TFSI_2	-87
Li_3TFSI_3	-146
Li_2TFSI_2 + DMSO → $[Li_2TFSI{\cdot}DMSO]^+$ + $TFSI^–$	+2.5
Li_3TFSI_3 + DMSO → $[Li_3TFSI_2{\cdot}DMSO]^+$ + $TFSI^–$	-0.8

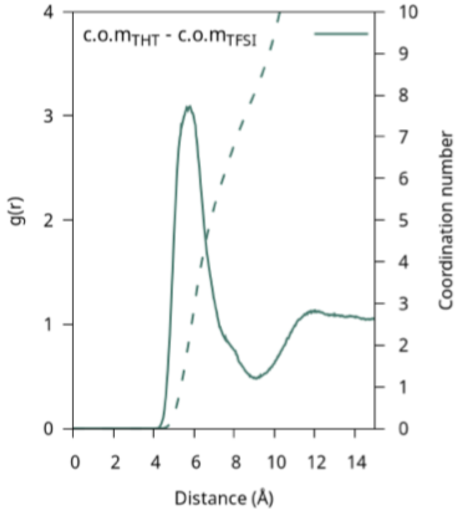

Figure S3. RDF and coordination number of the center of mass (c.o.m) of $TFSI^-$ and THT in the DMSO:THT solution.

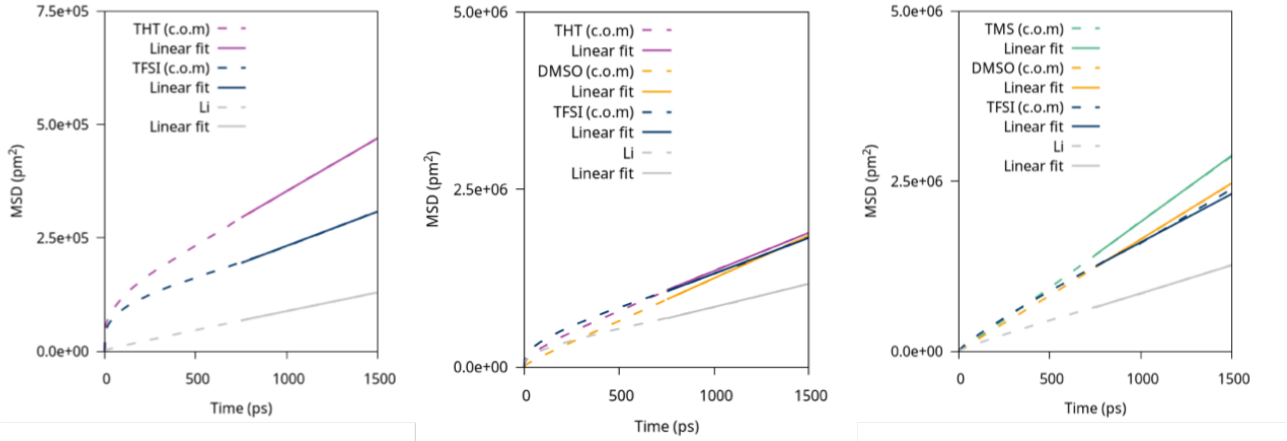

Figure S4: MSD plot with relative fitting for Li cation, TFSI anion, and solvent molecules for the three electrolyte solutions. The data are plotted using a dashed line, the linear fitting (over the last 2/3 of data points) with a solid line.

Table S3. Diffusion coefficients (D) from NMR and MD in 10^{-10} m^2 s^{-1}; Conductivities (σ) in mS cm^{-1} and Li^+ transport numbers (tn) from MD, NMR and EIS. These data have been computed using simulations where the LiTFSI salt was **entirely dissociated at the beginning of the simulation**.

	Specie	**D MD**	D NMR	**σ MD**	σ NMR	σ EIS	**tn MD**	tn NMR	tn EIS
THT:DMSO LiTFSI 1m	Li	**1.7**	0.8	**16.94**	9.59	4.41	**0.50**	0.39	0.55
	TFSI	**1.8**	1.2						
	THT	**4.9**	1.4						
	DMSO	**8.8**	1.7						
THT LiTFSI 1m	Li	**1.4**	0.3	**13.73**	3.74	2.30	**0.46**	0.37	0.48
	TFSI	**1.7**	0.5						
	THT	**6.6**	0.6						
TMS:DMSO LiTFSI 1m	Li	**2.2**	0.7	**32.72**	12.37	0.71	**0.46**	0.38	0.24
	TFSI	**2.6**	1.1						
	TMS	**9.7**	1.4						
	DMSO	**11.5**	1.4						

Force field parameters

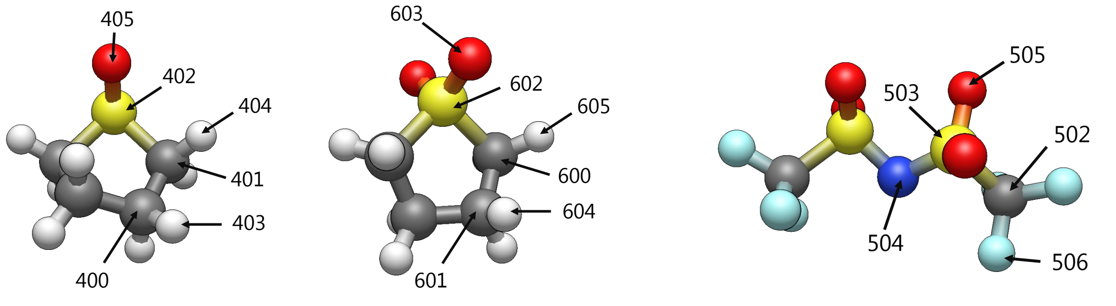

THT TMS TFSI

Figure S5: Chemical structure of THT and TMS solvent molecules, TFSI anion. After parametrization for the AMOEBA model, a specific atom type was assigned to each element.

Structures and topologies

THT

14

1	S	-0.95987649	0.13311161	-0.60214369	402	2	3	6	
2	O	-1.86307138	-0.05484994	0.60730302	405	1			
3	C	0.34328672	1.35890331	-0.07927537	401	1	4	7	8
4	C	1.67881857	0.60625387	0.08886623	400	3	5	9	10
5	C	1.29522951	-0.83280457	0.49237900	400	4	6	11	12
6	C	0.25151674	-1.25992257	-0.54515809	401	1	5	13	14
7	H	0.40162089	2.15446917	-0.82594368	404	3			
8	H	-0.00280145	1.79876778	0.85925770	404	3			
9	H	2.31084173	1.05630126	0.85827875	403	4			
10	H	2.25319781	0.58324761	-0.84118374	403	4			
11	H	0.85149895	-0.82455237	1.49101401	403	5			
12	H	2.17165423	-1.48526726	0.50621455	403	5			
13	H	0.66668936	-1.41814271	-1.54456467	404	6			
14	H	-0.28400318	-2.16243218	-0.24281702	404	6			

TMS

15

1	C	-1.755263	-0.762635	0.133023	601	2	3	8	9
2	C	-1.776635	0.750715	-0.131683	601	1	4	14	15
3	C	-0.435737	-1.268711	-0.442563	600	1	5	10	11
4	C	-0.440982	1.294758	0.374006	600	2	5	12	13
5	S	0.777305	0.000530	0.003283	602	3	4	6	7
6	O	1.554146	0.378932	-1.187379	603	5			
7	O	1.445368	-0.397329	1.252808	603	5			
8	H	-2.608882	-1.271764	-0.322750	604	1			
9	H	-1.788527	-0.951637	1.211652	604	1			
10	H	-0.461315	-1.322076	-1.534401	605	3			
11	H	-0.087303	-2.217845	-0.029745	605	3			
12	H	-0.109469	2.213904	-0.113680	605	4			
13	H	-0.437254	1.426505	1.459057	605	4			
14	H	-1.871870	0.932783	-1.207730	604	2			
15	H	-2.616671	1.244070	0.364932	604	2			

TFSI

15

1	C	-0.938434	0.547059	-1.836521	502	2	3	4	5
2	F	0.245910	0.881730	-2.397350	506	1			
3	F	-1.868124	1.387981	-2.355331	506	1			
4	F	-1.323200	-0.707765	-2.171151	506	1			
5	S	-0.975892	0.794758	0.027331	503	1	6	7	8
6	O	-0.315754	2.108071	0.121712	505	5			
7	O	-2.368901	0.670758	0.498122	505	5			
8	N	-0.024316	-0.339040	0.672657	504	5	9		
9	S	1.283909	-1.014101	0.012704	503	8	10	11	12
10	O	1.188645	-1.362206	-1.413195	505	9			
11	O	1.858123	-1.999719	0.948766	505	9			
12	C	2.472207	0.433668	0.131340	502	9	13	14	15
13	F	3.713350	-0.035907	-0.150798	506	12			
14	F	2.507742	0.936736	1.389420	506	12			
15	F	2.199204	1.403307	-0.767657	506	12			

Force field parameters

THT

```
atom          402    402    S     "Tetrahydrothiophene1-oxide"                    16    32.066    3
atom          405    405    O     "Tetrahydrothiophene1-oxide"                     8    15.999    1
atom          401    401    C     "Tetrahydrothiophene1-oxide"                     6    12.011    4
atom          400    400    C     "Tetrahydrothiophene1-oxide"                     6    12.011    4
atom          404    404    H     "Tetrahydrothiophene1-oxide"                     1     1.008    1
atom          403    403    H     "Tetrahydrothiophene1-oxide"                     1     1.008    1

vdw 401 3.8200 0.1010
vdw 400 3.8200 0.1010
vdw 402 3.9100 0.3850
vdw 404 2.9100 0.0330 0.900
vdw 405 3.4 0.1
vdw 403 2.96 0.024 0.92

bond 405 402 545.501115 1.52
bond 401 402 143.677779 1.83
bond 400 401 205.758598 1.53
bond 404 401 340.532535 1.09
bond 400 400 205.758598 1.53
bond 403 400 340.532535 1.09
bond 401 400 205.758598 1.53

angle 402   401   400      60.0     105.7978
angle 401   402   401      80.0      86.4166
angle 404 401 402 44.362438 109.4178
angle 401 402 405 97.668561 106.4000
angle 400 400 401 74.895704 105.9900
angle 403 400 401 48.260518 111.5502
angle 404 401 400 48.260518 111.7125
angle 401 400 400 74.895704 105.9900
angle 403 400 400 48.260518 109.3788
angle 404 401 404 30.490529 110.3853
angle 403 400 403 30.490529 109.1031

strbnd 402   401   400      0      0
strbnd 401   402   401      0      0
strbnd 404 401 402 5.7126 5.7126
strbnd 401 402 405 9.1453 9.1453
strbnd 400 400 401 5.7126 5.7126
strbnd 403 400 401 5.7126 5.7126
strbnd 404 401 400 5.7126 5.7126
strbnd 401 400 400 5.7126 5.7126
strbnd 403 400 400 5.7126 5.7126
strbnd 404 401 404 5.7126 5.7126
strbnd 403 400 403 5.7126 5.7126

torsion 401 400 400 401 0.854 0.0 1 -0.374 180.0 2 0.108 0.0 3
torsion 403 400 400 401 0 0.0 1 0 180.0 2 0.108 0.0 3
torsion 404 401 400 400 0 0.0 1 0 180.0 2 0.108 0.0 3
torsion 402 401 400 400 0.854 0.0 1 -0.374 180.0 2 0.108 0.0 3
torsion 402 401 400 403 0 0.0 1 0 180.0 2 0.108 0.0 3
torsion 404 401 400 403 0.000 0.0 1 0.000 180.0 2 0.299 0.0 3
torsion 403 400 400 403 0.000 0.0 1 0.000 180.0 2 0.299 0.0 3
torsion 405 402 401 400 0.854 0.0 1 -0.374 180.0 2 0.108 0.0 3
torsion 404 401 402 401 0 0.0 1 0 180.0 2 0.108 0.0 3
torsion 400 401 402 401 0.854 0.0 1 -0.374 180.0 2 0.108 0.0 3
torsion 404 401 402 405 0 0.0 1 0 180.0 2 0.108 0.0 3

polarize           402          2.9941     0.3900 405
polarize           405          0.8588     0.3900 402
polarize           401          1.6196     0.3900 404
polarize           400          1.4150     0.3900 403
polarize           404          0.4803     0.3900 401
polarize           403          0.4803     0.3900 400

multipole   402 -401 -401              0.97594
                                       0.00000    0.00000   -0.04113
                                       0.36284
                                       0.00000   -1.40207
                                       0.00000    0.00000    1.03923
multipole   405  402                  -0.69864
                                       0.00000    0.00000   -0.82862
                                       0.25555
                                       0.00000    0.25555
                                       0.00000    0.00000   -0.51110
multipole   401  400  402             -0.48258
```

```
                                        -0.24557    0.00000   -0.01898
                                        -0.49188
                                         0.00000   -0.10698
                                        -0.15255    0.00000    0.59886
multipole   400  401  400               -0.04681
                                         0.13934    0.00000    0.10609
                                         0.40982
                                         0.00000   -0.68284
                                         0.06725    0.00000    0.27302
multipole   404  401  404                0.10411
                                         0.05159    0.00000   -0.09596
                                         0.04775
                                         0.00000    0.02870
                                         0.32871    0.00000   -0.07645
multipole   403  400  403                0.09126
                                        -0.00530    0.00000   -0.26655
                                         0.11154
                                         0.00000    0.23125
                                         0.18587    0.00000   -0.34279
```

TMS

```
atom          601    601    C     “sulfolane”   6    12.011    4
atom          600    600    C     “sulfolane”   6    12.011    4
atom          602    602    S     “sulfolane”  16    32.066    4
atom          603    603    O     “sulfolane”   8    15.999    1
atom          604    604    H     “sulfolane”   1     1.008    1
atom          605    605    H     “sulfolane”   1     1.008    1


vdw 601 3.8200 0.1010
vdw 600 3.8200 0.1010
vdw 602 3.9100 0.3850
vdw 605 2.9100 0.0330 0.900
vdw 603 3.4 0.1
vdw 604 2.96 0.024 0.92


bond 603 602 606.0      1.47
bond 600 601 205.758598 1.53
bond 604 601 340.532535 1.09
bond 601 601 205.758598 1.54
bond 605 600 340.532535 1.09
bond 602 600 180.367537 1.81

angle 602   600   601      60.0     105.04
angle 603   602   600      75.0     109.24
angle 603   602   603      80.0     121.13
angle 605 600 601 48.260518 113.36
angle 604 601 601 48.260518 110.89
angle 600 601 601 74.895704 106.12
angle 604 601 600 48.260518 110.58
angle 600 602 600 92.59778 95.76
angle 605 600 602 47.74336 107.3
angle 604 601 604 30.490529 107.78
angle 605 600 605 30.490529 109.98

strbnd  602   600   601      0      0
strbnd 605 600 601 5.7126 5.7126
strbnd 604 601 601 5.7126 5.7126
strbnd 600 601 601 5.7126 5.7126
strbnd 604 601 600 5.7126 5.7126
strbnd 600 602 600 8.991 8.991
strbnd 605 600 602 5.7126 5.7126
strbnd 604 601 604 5.7126 5.7126
strbnd 605 600 605 5.7126 5.7126
torsion 600 601 601 600 0.854 0.0 1 -0.374 180.0 2 0.108 0.0 3
torsion 605 600 601 601 0 0.0 1 0 180.0 2 0.108 0.0 3
torsion 604 601 601 600 0 0.0 1 0 180.0 2 0.108 0.0 3
torsion 602 600 601 601 0.854 0.0 1 -0.374 180.0 2 0.108 0.0 3
torsion 602 600 601 604 0 0.0 1 0 180.0 2 0.108 0.0 3
torsion 605 600 601 604 0.000 0.0 1 0.000 180.0 2 0.299 0.0 3
torsion 604 601 601 604 0.000 0.0 1 0.000 180.0 2 0.299 0.0 3
torsion 603 602 600 601 0.854 0.0 1 -0.374 180.0 2 0.108 0.0 3
torsion 605 600 602 603 0 0.0 1 0 180.0 2 0.108 0.0 3
torsion 605 600 602 600 0 0.0 1 0 180.0 2 0.108 0.0 3
torsion 601 600 602 600 0.854 0.0 1 -0.374 180.0 2 0.108 0.0 3
```

```
polarize          601          1.4150     0.3900 604
polarize          600          1.6196     0.3900 605
polarize          602          2.9941     0.3900 603
polarize          603          0.8588     0.3900 602
polarize          604          0.4803     0.3900 601
polarize          605          0.4803     0.3900 600


multipole   601  600  601              -0.14032
                                        0.24618    0.00000    0.15048
                                        0.29194
                                        0.00000   -0.56773
                                       -0.15694    0.00000    0.27579
multipole   600  602  601              -0.33810
                                        0.23904    0.00000    0.09639
                                        0.52365
                                        0.00000   -0.36567
                                       -0.36450    0.00000   -0.15798
multipole   602 -600 -600               1.44112
                                        0.00000    0.00000   -0.40704
                                       -0.77178
                                        0.00000    0.53426
                                        0.00000    0.00000    0.23752
multipole   603  602                   -0.65992
                                        0.00000    0.00000   -0.06776
                                       -0.35677
                                        0.00000   -0.35677
                                        0.00000    0.00000    0.71354
multipole   604  601  604               0.08966
                                       -0.02882    0.00000   -0.04009
                                        0.00535
                                        0.00000    0.03016
                                       -0.01552    0.00000   -0.03551
multipole   605  600  605               0.11923
                                       -0.01921    0.00000   -0.00890
                                        0.01430
                                        0.00000    0.01659
                                        0.00146    0.00000   -0.03089
```

TFSI

```
atom        504  504    N     "Bistriflimide (TFSI)"                       7    14.007    2
atom        503  503    S     "Bistriflimide (TFSI)"                      16    32.066    4
atom        505  505    O     "Bistriflimide (TFSI)"                       8    15.999    1
atom        502  502    C     "Bistriflimide (TFSI)"                       6    12.011    4
atom        506  506    F     "Bistriflimide (TFSI)"                       9    18.998    1

vdw         502              3.8200         0.1010
vdw         504              3.7100         0.1100
vdw         506              3.22           0.061
vdw         505              3.4            0.1
vdw         503              3.91           0.385

bond        504  503         550.0          1.61
bond        503  502         250.0          1.84
bond        505  503         606.0          1.47
bond        506  502         348.980134     1.35

angle       504  503  505     75.0          111.53450000000001
angle       504  503  502     75.0           92.4879
angle       503  504  503     65.0          114.73199999999999
angle       503  502  506     60.0          108.24429999999998
angle       505  503  502     75.0          102.4452
angle       505  503  505     80.0          121.78380000000001
angle       506  502  506     89.148437     109.87589999999999

strbnd      504  503  505      0            0
strbnd      504  503  502      0            0
strbnd      503  504  503      0            0
strbnd      503  502  506      0            0
strbnd      506  502  506     5.7126       5.7126

torsion     506  502  503  504      1.500 0.0 1  -0.963 180.0 2    1.095 0.0 3
torsion     505  503  504  503     -9.798 0.0 1   5.169 180.0 2    6.577 0.0 3
torsion     502  503  504  503     -3.857 0.0 1   6.970 180.0 2  -12.118 0.0 3
torsion     506  502  503  505     -2.745 0.0 1   2.478 180.0 2   -0.220 0.0 3
```

```
multipole   504 -503 -503              -0.68562
                                        0.00000    0.00000    0.35319
                                        0.30120
                                        0.00000   -0.69514
                                        0.00000    0.00000    0.39394
multipole   503  502  504               1.29641
                                       -0.14077    0.00000    0.04086
                                       -0.06100
                                        0.00000    1.06990
                                        0.36904    0.00000   -1.00890
multipole   505  503  502              -0.69730
                                       -0.07142    0.00000    0.01967
                                       -0.25524
                                        0.00000   -0.28001
                                       -0.04944    0.00000    0.53525
multipole   502  503  504               0.59764
                                       -0.06173    0.00000   -0.28462
                                        0.40625
                                        0.00000    0.28817
                                       -0.14963    0.00000   -0.69442
multipole   506  502                   -0.21888
                                        0.00000    0.00000    0.15334
                                       -0.23731
                                        0.00000   -0.23731
                                        0.00000    0.00000    0.47462

polarize    504          1.4437     0.3900    503
polarize    503          2.9941     0.3900    504 505
polarize    505          0.8588     0.3900    503
polarize    502          1.6196     0.3900    506
polarize    506          0.3481     0.3900    502
```
